# Viperin-like proteins interfere with RNA viruses in plants

**DOI:** 10.3389/fpls.2024.1385169

**Published:** 2024-06-04

**Authors:** Radwa Kamel, Rashid Aman, Magdy M. Mahfouz

**Affiliations:** Laboratory for Genome Engineering and Synthetic Biology, Division of Biological Sciences, King Abdullah University of Science and Technology, Thuwal, Saudi Arabia

**Keywords:** viperin, viperin-like proteins, RNA virus interference, molybdenum cofactor synthesis 1A (MOCS1A), MoaA, CNX2

## Abstract

Plant viruses cause substantial losses in crop yield and quality; therefore, devising new, robust strategies to counter viral infections has important implications for agriculture. Virus inhibitory protein endoplasmic reticulum-associated interferon-inducible (Viperin) proteins are conserved antiviral proteins. Here, we identified a set of Viperin and Viperin-like proteins from multiple species and tested whether they could interfere with RNA viruses *in planta*. Our data from transient and stable overexpression of these proteins in *Nicotiana benthamiana* reveal varying levels of interference against the RNA viruses tobacco mosaic virus (TMV), turnip mosaic virus (TuMV), and potato virus x (PVX). Harnessing the potential of these proteins represents a novel avenue in plant antiviral approaches, offering a broader and more effective spectrum for application in plant biotechnology and agriculture. Identifying these proteins opens new avenues for engineering a broad range of resistance to protect crop plants against viral pathogens.

## Introduction

Viruses are considered the most abundant entities in the world that rely on the host organisms to replicate (Mateu, 2013; Koonin and Dolja, 2014). Both prokaryotic and eukaryotic cells are susceptible to viral infections, and as a response, antiviral mechanisms have been developed in both domains. These mechanisms aim to recognize the molecular signatures of the invading viruses and defend against them (Koonin, 2017; Tan et al., 2018). In response to viral infections, cells employ various strategies to protect themselves (Kawai and Akira, 2006; Koyama et al., 2008). Nevertheless, viruses can evolve mechanisms to counteract the immune system, impairing its effectiveness. As a result, cells are driven to innovate new strategies for combating viral threats. Hence, the immune system faces considerable evolutionary pressure, urging it to diversify in response (Goodbourn et al., 2000; Randall and Goodbourn, 2008). For this reason, distinct forms of immunity have evolved in prokaryotic and eukaryotic cells. Bacteria, for instance, rely on restriction-modification enzymes and the CRISPR-Cas system as a defensive system against viruses (Wilson and Murray, 1991; Horvath and Barrangou, 2010; Cong et al., 2013; Vasu and Nagaraja, 2013; Tesson et al., 2022). In contrast, eukaryotes employ alternate approaches like Nucleotide oligomerization and binding domain (NOD)-like receptors (NLRs) and RNAi interference, particularly in the case of plants (Agrawal et al., 2003; Franchi et al., 2006; Shabalina and Koonin, 2008; Wilmanski et al., 2008).

The restriction-modification system uses enzymes to detect and cut foreign DNA that lacks the correct methylation pattern. This mechanism can prevent the viral DNA from replication inside the host cell (Oliveira et al., 2016). On the other hand, the CRISPR-Cas system incorporates short viral genome fragments into the bacterial genome, creating a memory of the past infection (Bhaya et al., 2011; Datsenko et al., 2012). Consequently, whenever the cell reencounters a matching viral sequence, this system leverages its memory to identify and eliminate the invading viral nucleic acid (Cong and Zhang, 2015). RNA viruses, constituting the primary class of plant-infecting viruses are categorized into diverse groups and subgroups. These classifications are based on genetic similarities among key virus genes: movement protein (MP), coat protein (CP), and RNA-dependent RNA polymerase (RdRp), determined through phylogenetic connections (Murphy et al., 2012; Hull, 2013; Walker et al., 2021). Many of these viruses possess positive-sense, single-stranded RNA genomes that cause severe diseases in many agriculturally important crops (Budziszewska and Obrępalska-Stęplowska, 2018). Different types of CRISPR/Cas systems targeting RNA genomes have been used to combat RNA viruses in different hosts (Abudayyeh et al., 2016, 2017; Aman et al., 2018a, 2018b; Mahas et al., 2019). Although genetic engineering can develop and strengthen resistance against plant pathogens, despite its advantages, several limitations hinder its widespread applications (Sharma et al., 2004; Tatineni and Hein, 2023).

To overcome this, virologists and molecular biologists are actively seeking antiviral solutions that can effectively combat disease-causing viruses in animals and plants. Genetic manipulations offer a quick way of introducing plant resistance traits against specific pathogens, making them particularly valuable in combating viral diseases that arise unexpectedly (Dasgupta et al., 2003; Cao et al., 2020). Many methods to create virus-resistant plants rely on pathogen-derived resistance (PDR), where viral sequences are introduced into plant cells to provide protection against viruses (Goldbach et al., 2003). The PDR approach can be divided into two groups: nucleic acid-mediated resistance and protein-mediated resistance. Several viral proteins are used for this approach, like movement proteins, proteases, replicases, and coat proteins (Gottula and Fuchs, 2009). Additionally, the use of transgenic RNA instead of expressed viral proteins has created new opportunities for RNA-based resistance methods against viruses (Tenllado et al., 2004; Taliansky et al., 2021).

A significant finding from a recent study by *Aude Bernheim et al*. has revealed the ability of prokaryotic viperins to produce a set of modified ribonucleotides capable of hindering the replication of several viruses. Noteworthy, among these modified ribonucleotides are 3′-deoxy-3′,4′-didehydro (ddh)-cytidine triphosphate (ddhCTP), and ddh-guanosine triphosphate (ddhGTP) and ddh-uridine triphosphate (ddhUTP) (Bernheim et al., 2021). Viperins are evolutionarily conserved and induced by interferons (Rivera-Serrano et al., 2020). In humans, viperins demonstrate robust antiviral activity, effectively combating both RNA and DNA viruses such as hepatitis C virus, HIV, dengue virus, West Nile virus, and human cytomegalovirus (Gizzi et al., 2018; Rivera-Serrano et al., 2020). Moreover, Viperins are also known as radical S-adenosyl-L-methionine (SAM) domain containing 2 (RSAD2) due to the presence of this motif. Enzymes within the radical SAM superfamily (RS family) are found across all kingdoms of life. These enzymes are characterized by the presence of a highly conserved RS motif CX3CX2C (residues 83-90) and utilizing SAM as a cofactor (Duschene and Broderick, 2010; Shaveta et al., 2010). Viperins and enzymes in the radical S-adenosyl-L- methionine superfamily, particularly the MoaA enzyme, which is involved in the molybdenum cofactor biosynthesis, show notable similarity in their central domain (Boudinot et al., 1999; Chin and Cresswell, 2001). Moco biosynthesis bas been characterized in diverse organisms such as humans, *Arabidopsis, Aspergillus nidulans*, and bacteria (Schwarz and Mendel, 2006). It begins with the condensation of GTP to form cyclic pyranopterin monophosphate (cPMP). This process involves the combined action of GTP 3’,8 -cyclase and cPMP synthase, encoded by *CNX2* and *CNX3* in plants, and their bacterial homologs *MoaA* and *MoaC*, or human counterparts *MOCS1A* and *MOCS1B* (Mendel and Schwarz, 2011; Mendel, 2013; Hover et al., 2015). Radical SAM enzymes, found across all kingdoms of life, facilitate a remarkable array of complex and chemically challenging reactions (Frey et al., 2008). The broad-spectrum antiviral activity demonstrated by members of the radical SAM family against RNA viruses positions these proteins as highly promising candidates for novel antiviral therapeutic approaches (Helbig and Beard, 2014).

In this study, we investigated the antiviral efficiency of Viperin and Viperin-like proteins for RNA virus interference in *Nicotiana benthamiana*. Our approach involved a comprehensive analysis of known Viperin proteins through sequence homology searches, identifying similar proteins and testing them for virus interference in plants. Our data from transient and stable line experiments highlighted the distinct antiviral roles played by these proteins against RNA viruses in planta. Specifically, we identified that molybdenum cofactor biosynthetic enzyme MoaA from different species demonstrated interference against plant RNA viruses. Our findings not only contribute to a deeper understanding of the molecular mechanisms underlying the antiviral activity of these proteins but also position them as promising tools for adoption as antiviral strategies in planta. Notably, our identified set of antiviral proteins offers a streamlined and efficient approach, contrasting with CRISPR, where the design of specific crRNA for individual virus targeting is imperative. This discovery streamlines the process, presenting our findings as a versatile solution with broad-spectrum antiviral benefits, showcasing potential advantages over current technologies.

## Results

### Identification, design, build and test genetic constructs of Viperin-like proteins for viral interference in planta

**Figure 1:**
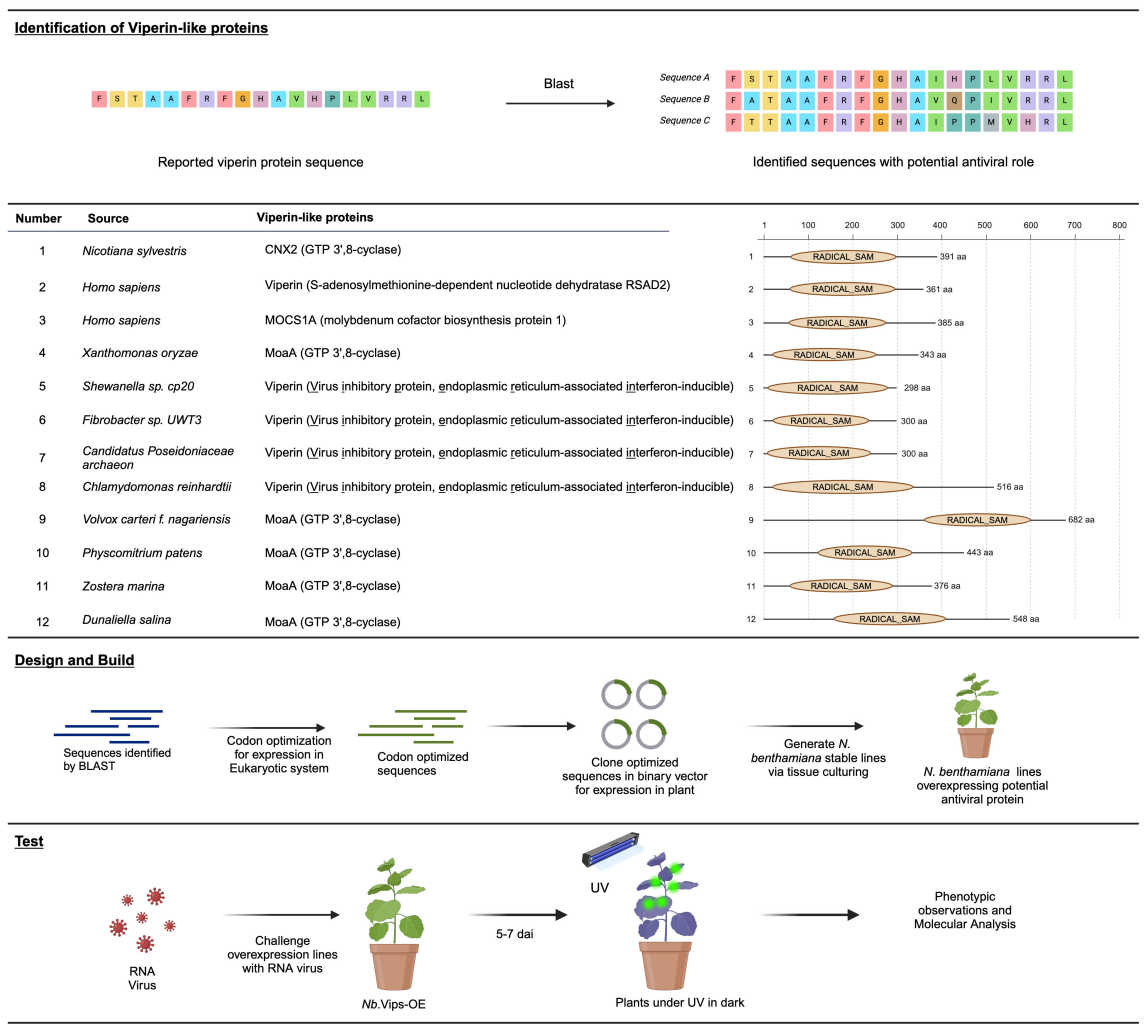
Exploration of potential antiviral proteins for RNA virus interference in planta. In the upper panel, an online search unveils reported and predicted VIPERINS and their homologs. The middle panel list potential antiviral proteins and their homologs, identified through a detailed blast search. To optimize coding sequences for expression in eukaryotic systems, they were cloned into a binary vector under the control of a constitutive promoter. Subsequently, the binary vector was utilized to generate stable N. benthamiana lines overexpressing the potential antiviral proteins through tissue culturing. Plants challenged with virus were analyzed under UV light in the dark. Leaf samples were collected and the whole transcriptomic analysis was performed and the data was analyzed.

Viperins, a class of proteins, have been reported to confer an inhibitory strategy against viruses in eukaryotes and prokaryotes. Structural alignments have indicated that Viperin shares structural similarities with radical SAM proteins, including the molybdenum cofactor biosynthetic enzyme MoaA (Fenwick et al., 2017). Building on these findings, we selected Viperins from *Homo sapiens* and other organisms, as well as MoaA proteins from different species, to assess their antiviral activity in plants. Considering their prevalence across diverse organisms, we chose Viperins and Viperin-like proteins from bacteria, algae, plants, and archaea to be tested in our study. Our selection of protein sequences was based on the work done by Audi Bernheim et al. while others were chosen based on their homology to the selected proteins as identified by the BLAST analysis (**Figure 1**; **Supplementary Figure 1**; **Supplementary Table 1**). The identified sequences were codon-optimized for expression in the eukaryotic system and cloned into a binary vector for subsequent expression in planta. Subsequently, we generated *N. benthamiana* stable lines through tissue culture and confirmed the presence of protein via western blot (**Supplementary Figure 2**). The confirmed lines were challenged with GFP-tagged plant RNA viruses TMV-GFP, TuMV-GFP and PVX-GFP. Plants were observed for GFP fluorescence under UV 5 to 7 days after infiltration. Following phenotypic analysis, systemic leaf samples were harvested for any downstream molecular analysis.

### Transient assays reveal that viperin and viperin-like proteins have activity against TMV

To expedite the screening of candidate Viperin and Viperin-like proteins, we first conducted transient assays using a tobacco mosaic virus (TMV)-based overexpression system, known as the TRBO-G system, which expresses the GFP gene as a reporter (Lindbo, 2007). In the TRBO-G system, the TMV has been engineered to replace the CP encoding sequence with a sequence encoding green fluoresent protein (GFP). The TRBO-G virus cannot move systematically in the infected plants but can efficiently replicate and produce GFP in the infected leaves. The utilization of this reporter system provided an easy and straightforward means to monitor virus infection and replication, thus accelerating the screening process.

**Figure 2:**
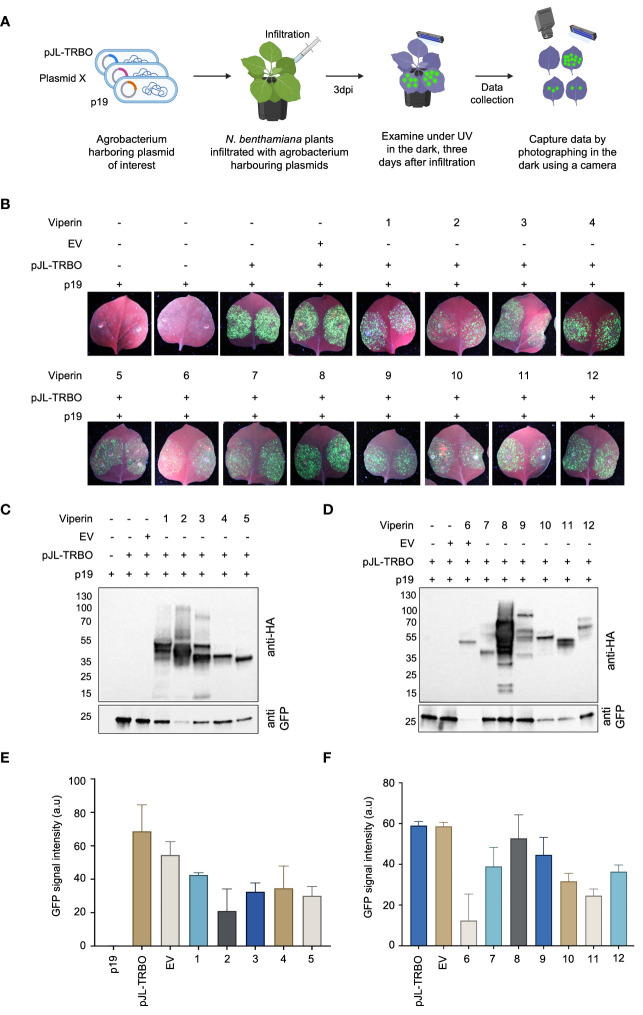
Assessment of diverse antiviral proteins for viral interference in planta **(A)** Schematic representation of the screening process of the selected antiviral proteins from different species. All reagents (pJL-TRBO plus Viperine) were delivered agrobacterium-mediated transiently into *N.benthamiana* leaves. Plants were checked for any green fluorescence three days after infiltration (3dai) under UV light, and photographs were taken in the dark with a Nikon digital camera. **(B)** GFP monitoring to assess the interference activity of the selected antiviral proteins in Agro-infiltrated wild-type *N. benthamiana* leaves in transient assays. Images were taken 3 days post-infiltration. EV represents an empty vector. **(C)** Western blot analysis of the abundance of the virus-expressed GFP protein to confirm the potentially identified antiviral proteins viral interference. Protein blots were developed with anti-HA antibody to detect viperin (upper panel) and anti-GFP antibody (lower panel) to detect the viral abundance due to viperine activity. The gel shows viperine proteins from 1 to 5. **(D)** The gel image shows the antiviral activity of potential viperin proteins from 6 to 12. The rest of the description remains the same as panel **(C)**. **(E)** Graphical representation of the quantification of the GFP abundance for protein 1 to 5. The intensity of the GFP-band was measured using image J and the values were plotted in Prism. Error bars indicate SEM (n = 3). **(F)** Graphical representation of the quantification of the GFP abundance for protein 1 to 6. The intensity of the GFP-band was measured using image J, and the values were plotted in Prism. Error bars indicate SEM (n = 3).

To assess the interference activity of Viperin and Viperin-like proteins, we co-delivered binary vector harboring a codon-optimized coding region for the selected protein and the TRBO-G expressing construct (pJL-TRBO) into the leaves of wild-type *N.benthamiana* plants via Agro-infiltration (**Figure 2A**). Plants infiltrated with TRBO-G alone or TRBO-G in combination with an empty binary vector (EV) were used as control groups. At 3 dpi, we observed varied levels of interference among the identified proteins when challenged with the TMV virus (**Figure 2B**). Our data indicated a significant reduction in GFP signals for Viperin (from *Homo sapiens*), Molybdenum cofactor biosynthesis protein 1 MOCS1A (from *Homo sapiens*), Viperin (from *Shewanella* sp. *cp20*), Viperin (from *Fibrobacter* sp. *UWT3*), MoaA (from *Physcomitrium patens*), and MoaA (from *Zostera marina*) compared to the control, providing strong evidence of their antiviral activity against TMV in plants.

To validate the observed reduction in GFP fluorescence, we measured GFP protein in the infiltrated leaves by western blot. Total proteins were extracted from the leaves, and western blot was performed using anti-HA and anti-GFP antibodies to detect Viperin and Viperin-like proteins and the virus protein, respectively. In agreement with the observed low GFP fluorescence, GFP protein levels were also suppressed by some of the Viperin and Viperin-like proteins (**Figures 2C, D**). Notably, Viperin from *Homo sapiens*, and Viperin from *Fibrobacter* sp. *UWT3* exhibited robust antiviral activity against TMV, as indicated by the presence of less GFP protein. The graphical representation of the GFP protein abundance further corroborates our observation (**Figures 2E, F**). These data indicate that Viperin and Viperin-like proteins exhibit an antiviral role against plant viruses *in planta*.

### The molybdenum cofactor biosynthetic enzyme MoaA from different species confer resistance to TuMV in plants

To explore the potential of antiviral proteins in conferring heritable resistance against RNA viruses in plants, we created transgenic *N. benthamiana* plants heterologously overexpressing Viperin and Viperin-like proteins driven by the cauliflower mosaic virus *35S* promoter. Protein presence was validated through western blot analysis, and the confirmed lines were used in the following experiments (**Supplementary Figure 2**).

**Figure 3:**
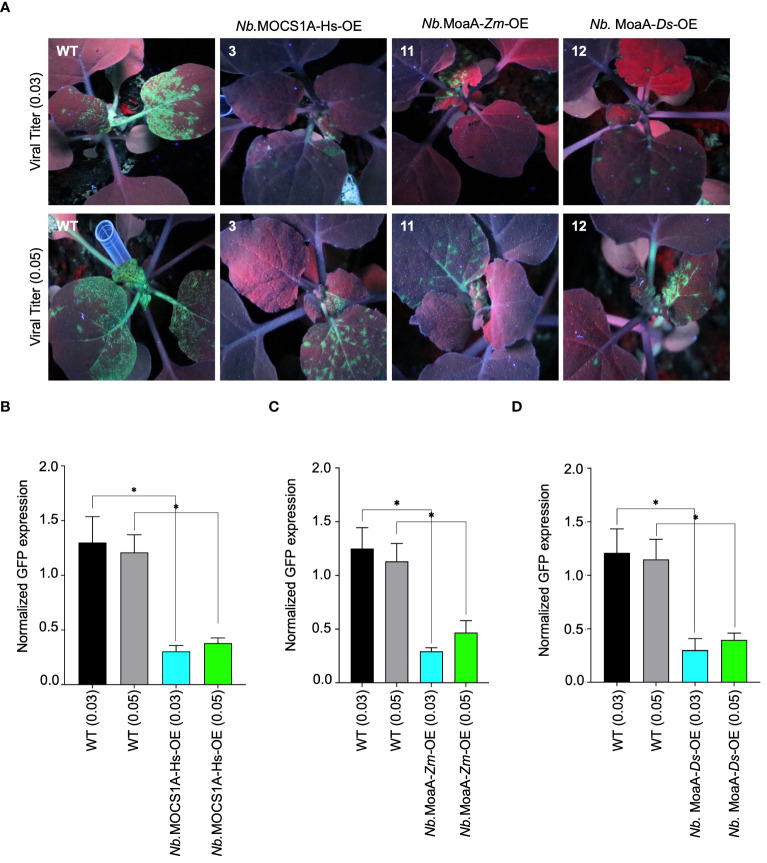
Antiviral role of molybdenum cofactor biosynthetic enzyme from different species against TuMV-GFP in N. *benthamiana* plants stable lines. **(A)** Radicle SAM proteins exhibits interference against TuMV-GFP in transgenic tobacco lines. *N. benthamiana* lines stably expressing Viperin-like proteins were agroinfiltrated with TuMV-GFP virus. Plants were imaged for GFP fluorescence to examine TuMV-GFP systematic spread under UV light in the dark 5-7 dai. The *N. benthamiana* plants Nb.MOCS1A-Hs-OE overexpresses MOCS1A from *Homo sapiens*, *Nb*.MoaA-Zm-OE overexpresses MoaA from *Zostera marina* and *Nb*.MoaA-Ds-OE overexpresses MoaA from *Dunaliella salina*. **(B)** RT-qPCR analysis to quantify TuMV-GFP RNA in MOCS1A (*Homo sapiens*) overexpressing plants. For each protein, the interference efficiency is shown compared to the controls. **(C)** RT-qPCR analysis to quantify TuMV-GFP RNA in MoaA (*Zostera marina*) overexpressing plants. For each protein, the interference efficiency is shown compared to the controls. **(D)** RT-qPCR analysis to quantify TuMV-GFP RNA in MoaA (*Dunaliella salina*) overexpressing plants. For each protein, the interference efficiency is shown compared to the controls. The Student’s *t*-test analysis indicated a significant difference compared with the WT (*P<0.05). Values are the means of three biological repeats.

We then chose the potyvirus turnip mosaic virus (TuMV) as our target RNA virus and evaluated its pathogenicity on our stable plant lines. T2-generation data revealed varying interference levels among the tested lines challenged with TuMV-GFP (**Supplementary Figure 3**). Notably, *N. benthamiana* lines overexpressing molybdenum cofactor biosynthesis protein A (MOCS1A) from *Homo sapiens*, GTP 3’,8-cyclase (MoaA) from *Zostera marina* and *Dunaliella Salina* exhibited interference against TuMV-GFP. The identified resistant lines were then challenged with two TuMV-GFP titers (0.03 and 0.05 OD, based on the OD_600_ of the TuMV-carrying Agrobacteria used for inoculation). The results confirmed the resistance of these lines, as indicated by the reduced GFP fluorescence signal compared to the non-transgenic line (**Figure 3A**). RT-qPCR further confirmed the accumulation of less TuMV-GFP genomic RNA in the transgenic lines (**Figures 3B–D**). Additionally, a difference compared to the control was observed even when the viral titer was increased to 0.05, further confirming the resistance of these lines against TuMV-GFP.

To ensure there is no interference from the 35s promoter between different constructs, we tested our resistant lines using sap from wild-type plants infected with the TuMV-GFP virus. Despite using sap, our lines continued to exhibit resistance, indicating that the observed resistance is due to Viperin-like proteins and not because the 35s promoter in the transgenic lines is silencing the viral construct. Moreover, using a non-specific control supports the fact that the resistance observed is indeed due to Viperin-like proteins (**Supplementary Figure 4**).

### The molybdenum cofactor biosynthetic enzyme MoaA from different species confer resistance to PVX in plants

To evaluate the effectiveness of Viperin and Viperin-like proteins in interfering with other RNA viruses, we challenged our *N. benthamiana* stable lines with potato virus x (PVX) (**Supplementary Figure 5**). Like TuMV-GFP, we used a GFP-tagged PVX virus to facilitate visual detection. Our data from the T2 generation revealed varied levels of viral interference among the tested lines. *N. benthamiana* lines overexpressing CNX2 from *Nicotiana sylvestris*, MOCS1A from *Homo sapiens* and MoaA from *Xanthomonas oryzae* showed strong interference with PVX compared to the non-transgenic and empty vector (EV) controls.

**Figure 4:**
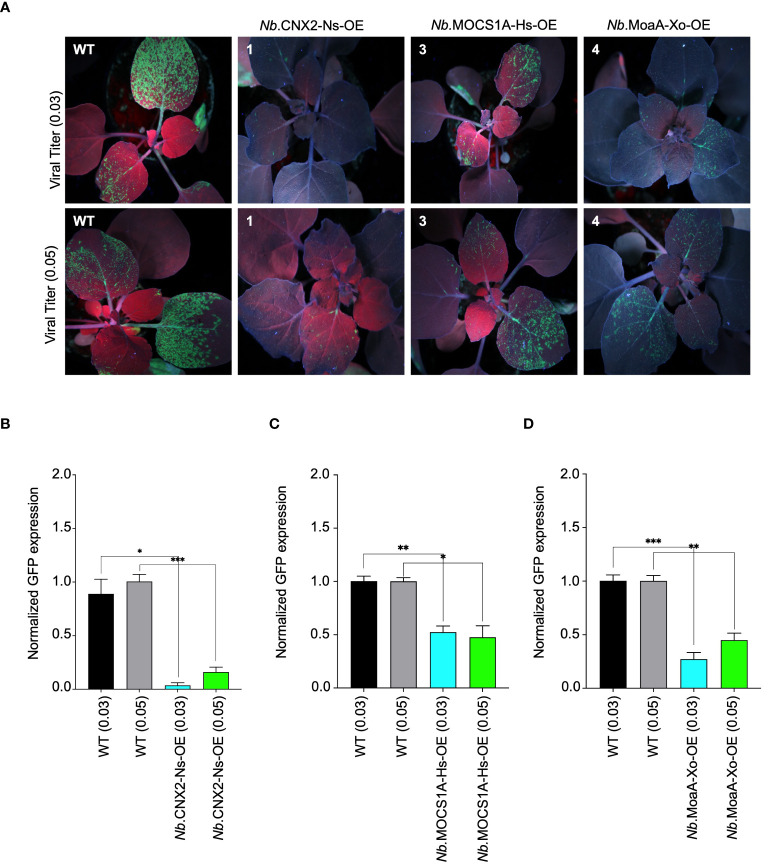
Antiviral role of molybdenum cofactor biosynthetic enzyme from different species against PVX-GFP in N. *benthamiana* plants stable lines. **(A)** Interference of *PVX-GFP* by radical SAM proteins expressed in tobacco transgenic lines. Stably transformed *N. benthamiana* lines expressing Viperin-like proteins were agroinfiltrated with two viral titer (OD 0.03 and 0.05) of *PVX-GFP* virus. Plants were imaged for GFP fluorescence to examine *PVX-GFP* systematic spread under UV light in the dark 5 to 7 days post infiltration. The *N. benthamiana* plants *Nb*.CNX2-Ns-OE overexpresses CNX2 from *Nicotiana Sylvesteris*, *Nb*.MOCS1A-Hs-OE overexpresses MOCS1A from *Homo sapiens*, and *Nb*.MoaA-Zm-OE overexpresses MoaA from *Xanothomonas oryzae.* **(B)** RT-qPCR analysis to quantify PVX-GFP RNA in cPMP-synthase overexpressing *N.benthamiana* plants. For each protein, the interference efficiency is shown compared to the controls. **(C)** RT-qPCR analysis to quantify PVX-GFP RNA in MOCS1 overexpressing *N.benthamiana* plants. For each protein, the interference efficiency is shown compared to the controls. **(D)** RT-qPCR analysis to quantify PVX-GFP RNA in MoaA overexpressing *N.benthamiana* plants. For each protein, the interference efficiency is shown compared to the controls. The Student’s *t*-test analysis indicated a significant difference compared with the WT (*P<0.05, **P<0.001, ***P<0.0009). Values are the means of three biological repeats.

To validate our findings, the identified resistant lines were challenged with two titers of PVX-GFP (0.03 and 0.05, based on the OD_600_ of the Agrobacteria) (**Figure 4A**). A visual decrease in GFP fluorescence observed in *N. benthamiana* lines overexpressing the mentioned proteins confirmed their role in virus interference. Notably, a strong reduction in GFP signal was observed in plants overexpressing CNX2 from *Nicotiana sylvestris*, indicating robust interference provided by this protein. Substantiating these findings, RT-qPCR analysis also showed a prominent decrease in PVX-GFP accumulation in these plants, especially those overexpressing CNX2. This aligns well with the observed phenotypic data (**Figures 4B–D**). We further confirmed our transgenic lines for resistance by challenging them with sap from wild-type plants previously infected with PVX-GFP and found that our lines remained resistant. The data confirms that the observed resistance is only due to Viperin-like proteins and not from the cross-interference mechanism, if any. Non-specific control further confirmed the interference of Viperin-like proteins (**Supplementary Figure 6**).

## Discussion

Viruses pose a significant threat to crop plants, prompting scientists to devise various strategies for combatting these infections (Tatineni and Hein, 2023). However, the limitations of existing approaches drive an ongoing search for a simplified and robust way to enhance plant resistance against viral threats (Jones and Naidu, 2019; Giudice et al., 2021). Here, we explored the antiviral activity of Viperin and Viperin-like proteins, specifically members of the radical S-adenosyl-methionine (SAM) enzyme family, in plants. We selected a total of 12 Viperin and Viperin-like proteins from various species, including bacteria, algae, plants, and archaea and assessed their ability to interfere with RNA viruses in *N. benthamiana* plants.

To evaluate the antiviral activity of the selected proteins, we used a transient assay exploiting the tobacco mosaic virus (TMV)-based overexpression system (TRBO-G system). This system allowed the quick assessment of proteins by measuring virus abundance in plant leaves through GFP expression. Our results demonstrated a significant reduction in GFP fluorescence signals when specific proteins were present compared to control groups. This data indicates the antiviral role of these Viperin and Viperin-like proteins against TMV in plants. We further investigated the potential of these antiviral proteins to confer heritable resistance against other RNA viruses, focusing on the potyvirus turnip mosaic virus (TuMV). Transgenic *N. benthamiana* plants overexpressing MoaA from different sources interfered with TuMV-GFP, as evidenced by reduced GFP fluorescence and decreased accumulation of TuMV-GFP genomic RNA.

To ensure broad-range interference against RNA viruses, our investigation extended to assess the proteins’ potential involvement in conferring resistance to another RNA virus, potato virus x (PVX). Similar to the TuMV results, *N. benthamiana* plants overexpressing CNX2, MOCS1A and MoaA displayed interference with PVX-GFP, indicating a broad-spectrum antiviral activity of these Viperin-like proteins. Our sap inoculation experiment provides further evidence that the observed virus interference is exclusively mediated by Viperin-like proteins, with no indication of other interference mechanisms at play.

Our findings show that molybdenum cofactor biosynthetic enzymes from different species possess broad antiviral activity against RNA viruses in plants. Particularly, MOCS1A from *Homo sapiens* demonstrated a broad-spectrum interference against two distinct RNA viruses, emphasizing the need for further research into the molecular mechanisms that enable this protein to combat multiple RNA viruses. Overexpressing MoaA might lead to alterations in secondary metabolites, potentially affecting the production of antiviral compounds. These alterations could influence plant immune responses, activating defense pathways and modulating gene expression linked to antiviral defense. Additionally, MoaA overexpression could activate stress responses that may enhance the plant’s resilience against viral challenges. The specific interactions between Moco-dependent enzymes and viral components during MoaA overexpression could impact viral replication, either directly or indirectly.

Viperins are recognized for their antiviral activity against both RNA and DNA viruses in animals (Helbig and Beard, 2014; Rivera-Serrano et al., 2020). While inherently absent in plants, the overexpression of Viperins in plant systems holds the potential to elicit diverse effects on plant defense mechanisms and cellular processes. This may involve the modulation of the plant’s immune response through the activation or enhancement of defense pathways crucial for virus recognition and combating. Although we did not observe any visible phenotypic changes in plants overexpressing Viperin and Viperin-like proteins, their overexpression might stimulate the activation of numerous stress-related genes. Further explorations into the metabolome profile of plants overexpressing Viperins during virus infection may reveal further insights into the antiviral role of these proteins in plants. Moreover, as part of our future work, we plan to assess the antiviral activity of the identified Viperin and Viperin-like proteins against DNA viruses.

Our study has shed light on the antiviral capabilities of different Viperin and Viperin-like proteins in plants against different plant-infecting RNA viruses and provides an efficient antiviral platform that can be potentially adopted to confer resistance to a range of viruses without the need to develop a virus-specific platform. Our work paves the way for future investigations by investigating molecular mechanisms and pathways, potentially leading to innovative methods for supporting broad-rang plant resistance against viral infections.

## Materials and methods

### Construction of viperin and viperin-like proteins for in planta expression

To engineer plants expressing Viperin and Viperin-like proteins, we mammalian codon-optimized their coding sequences for expression in the eukaryotic system and ordered them in intermediate vectors with a 3x-HA tag fused to the C-terminus of the sequence. All sequences were ordered as flanked by *attL1* and *attL2* recombination sites to facilitate gateway cloning. Cloning into the gateway binary vector pK2GW7 was performed by LR recombination to generate final clones harbouring Viperin and Viperin-like sequences driven by the cauliflower mosaic virus (CaMV) 35S promoter. Subsequently, the clones were introduced into *Agrobacterium tumefaciens* GV3101 via electroporation. Next, *N.benthamiana* leaf discs were treated with the agrobacterium cultures harboring the plasmids of interests and placed on regeneration media. Shoots from the regeneration media were then transferred to rooting media for root induction. The resulting plantlets were then transferred to soil, and the protein expression was confirmed by western blotting using anti-HA antibody. The confirmed lines were used in the subsequent experiments.

### Plant material

Two- to three-week-old wild-type *Nicotiana benthamiana* plants, cultivated under long-day conditions (16 hours light, 8 hours dark at 25°C), served as the experimental subjects for all transient assays. In transient experiments, wild-type *Nicotiana benthamiana* plants were initially grown on MS (Murashige & Skoog) media for 10 days and subsequently transplanted into soil. In experiments utilizing permanent lines (which stably expresses Viperin and Viperin-like proteins), T2 generation seeds were germinated on MS media supplemented with kanamycin, and the surviving seedlings were used in virus-targeting experiments.

### Agro-infiltration of *N.benthamiana* leaves and GFP imaging

The constructs containing pJL-TRBO (TMV-GFP), TuMV-GFP, and PVX-GFP were separately introduced into *Agrobacterium tumefaciens* strain GV3101 through electroporation. Single colonies grown overnight on a selective medium were centrifuged and resuspended in an infiltration medium (10 mM MES [pH 5.7], 10 mM CaCl2, and 200 μM acetosyringone) and incubated at ambient temperature for 2 hours. Infiltration into plants overexpressing Viperin and Viperin-like proteins involved using a cell density of 0.03 or 0.05 (OD600) for PVX-GFP and TuMV-GFP. A cell density of 0.005 was used in case of pJL-TRBO. The infiltration was performed on the abaxial side of leaves using a needle-less syringe. GFP expression was examined at 5-7 days after infiltration (dai) using a handheld UV light, and photographs were captured with a Nikon camera under UV light. Leaf samples were collected at 7 and 10 dai for subsequent molecular and transcriptomic analyses.

### Sap inoculation

To perform sap inoculation, 3-week-old wild-type *Nicotiana benthamiana* plants were infiltrated with Agrobacterium strain GV3101 carrying infectious clones of either TuMV-GFP or PVX-GFP. After 7 to 10 dpi (days post infiltration), systemic leaves were harvested and effectively crushed in phosphate buffer with a pH of 7.4 to collect sap. Next, individual leaves from 3-week-old wild-type *N. benthamiana* plants and transgenic lines overexpressing Viperin or Viperin-like proteins were dusted with 200–450 mesh-sized carborundum particles and applied with equal amount of sap containing either TuMV-GFP or PVX-GFP. Virus was spread uniformly on the leaf surface and the plants were kept in a greenhouse for another 7 to 10 days. To monitor virus spread, the plants were examined in darkness using a handheld ultraviolet (UV) light, and photographs were taken to record the results.

### Immunoblot analysis

Total proteins were extracted from 100 mg of the sample using an extraction buffer (100 mM Tris-Cl, pH 8, 150 mM NaCl, 0.6% IGEPAL, 1 mM EDTA, and 3 mM DTT) supplemented with protease inhibitors (PMSF, leupeptin, aprotinin, pepstatin, antipain, chymostatin, Na2VO3, NaF, MG132, and MG115). The protein extracts were separated on a 10% polyacrylamide gel. Immunoblot analysis was performed using a mouse α-GFP antibody (1:3000, Invitrogen) for GFP expressed by the virus and a rat α-HA (1:1000) antibody to detect viperin-like proteins. Antigens were visualized through chemiluminescence using an ECL-detecting reagent (Thermo Scientific). Quantitative analysis was conducted by calculating densitometric data from the relative quantification of protein bands on immunoblot membranes using ImageJ software, and the average values from three independent biological replicates were graphically summarized.

### RNA extraction and RT-qPCR for analysis of viral RNA genomes

Total RNA was extracted from systemic leaves using Direct-zol RNA Miniprep Kits (Zymo Research) following the manufacturer’s instructions. Viral RNA quantification was conducted through one-step RT-qPCR employing the iTaq Universal SYBR Green One-Step Kit (Bio-Rad). RT-qPCR reactions were executed in 10µl volumes using primers amplifying the GFP transcript (**Supplementary Table 2**) with three technical replicates in a 96-well format and analyzed using a StepOnePlusTM Real-Time PCR System (Applied Biosystems) for the 96-well plates. To normalize for total input, expression levels were determined by subtracting the cycle threshold (Ct) values of the housekeeping reference gene (tobacco PP2A) from the target Ct values, yielding ΔCt levels. The relative transcript abundance was calculated as 2−ΔΔCt. All analyses were performed with three biological replicates.

## Data availability statement

The original contributions presented in the study are included in the article/**Supplementary Material**. Further inquiries can be directed to the corresponding author.

## Author contributions

MM: Writing – review & editing, Supervision, Resources, Project administration, Conceptualization. RK: Writing – original draft, Methodology, Investigation. RA: Writing – review & editing, Writing – original draft, Validation, Supervision, Methodology, Investigation, Formal analysis.
