# Supplementary material for: Viperin-like proteins interfere with RNA viruses in plants
Source: Front Plant Sci. 2024 Jun 4;15:1385169. doi: 10.3389/fpls.2024.1385169 (PMC11185175; doi:10.3389/fpls.2024.1385169)
Supplement: Supplementary file 1 [file DataSheet_1.docx]

**Supplementary Data**

**Viperin-like proteins interfere with RNA viruses in plants**

Radwa Kamel^1^, Rashid Aman^1^ and Magdy M. Mahfouz^1^*

*^1^Laboratory for Genome Engineering and Synthetic Biology, Division of Biological Sciences, 4700 King Abdullah University of Science and Technology, Thuwal 23955-6900, Saudi Arabia.*

*Correspondence: Magdy M. Mahfouz ([magdy.mahfouz@kaust.edu.sa](mailto:magdy.mahfouz@kaust.edu.sa))

MoaA-Xanthomonas ------------------------------------------------------------ 0

MoaA-Volvox carteri MTRRSFVSRLTRYAIDCSCAKGPLNPSWPPQGAVGTHHVCCHAGGHEPSTSYAYGIQSTG 60

MoaA-Dunaliella sal ------------------------------------------------------------ 0

MOCS1A-Homo sapiens ------------------------------------------------------------ 0

MoaA-Physcomitrium ------------------------------------------------------------ 0

MoaA-Zostra marina ------------------------------------------------------------ 0

CNX2-Nicotiana. ------------------------------------------------------------ 0

Viperin-Shewanella ------------------------------------------------------------ 0

Viperin-Fibrobacter ------------------------------------------------------------ 0

Viperin-Homo sapiens ------------------------------------------------------------ 0

Viperin-Candidatus ------------------------------------------------------------ 0

Viperin-Chlamydomonas ------------------------------------------------------------ 0

MoaA-Xanthomonas ------------------------------------------------------------ 0

MoaA-Volvox carteri FHSVARRPSCARGFCSWPLTAAATSQTQSQEGNSSANWVLDPASAAEYDRRLREFLNASR 120

MoaA-Dunaliella sal -------------------------------------------------------MLQMR 5

MOCS1A-Homo sapiens ------------------------------------------------------------ 0

MoaA-Physcomitrium ------------------------------------------------------------ 0

MoaA-Zostra marina ------------------------------------------------------------ 0

CNX2-Nicotiana ------------------------------------------------------------ 0

Viperin-Shewanella ------------------------------------------------------------ 0

Viperin-Fibrobacter ------------------------------------------------------------ 0

Viperin-Homo sapiens ------------------------------------------------------------ 0

Viperin-Candidatus ------------------------------------------------------------ 0

Viperin-Chlamydomonas ------------------------------------------------------------ 0

MoaA-Xanthomonas ------------------------------------------------------------ 0

MoaA-Volvox carteri ALQDLPTARRDPPAPPPPPRPNRPLPPPPFHSFIAPAAA------ATIPAQDNTGFLVGD 174

MoaA-Dunaliella sal SLERLAISLRA-------FRQSASCSPPSWAAAPCASDIRGVQSDGDIGAPSSSGRDAGG 58

MOCS1A-Homo sapiens ----------------------------MWKSWKLRTDVRVREG---------------- 16

MoaA-Physcomitrium ---------------------------MRWVQWVALSSLRAARSLTTSSAPTGFG----- 28

MoaA-Zostra marina ------------------------------------------------------------ 0

CNX2-Nicotiana ------------------------------------------------------------ 0

Viperin-Shewanella ------------------------------------------------------------ 0

Viperin-Fibrobacter ------------------------------------------------------------ 0

Viperin-Homo sapiens ------------------------------------------------------------ 0

Viperin-Candidatus ------------------------------------------------------------ 0

Viperin-Chlamydomonas ------------------------------------------------------------ 0

MoaA-Xanthomonas ------------------------------------------------------------ 0

MoaA-Volvox carteri VATAVAAVASESTGSPAAASPTSPVPSTTSPPPGISPASPLSPPSLQSSSRVGLGPPEVA 234

MoaA-Dunaliella sal THQNWTLTWALRQGSPGTANSN-----TQDDRPWF----------------LRLEPSTAR 97

MOCS1A-Homo sapiens ------------------------------------------------------------ 16

MoaA-Physcomitrium ------------------------------------------------------------ 28

MoaA-Zostra marina ------------------------------------------------------------ 0

CNX2-Nicotiana ------------------------------------------------------------ 0

Viperin-Shewanella ------------------------------------------------------------ 0

Viperin-Fibrobacter ------------------------------------------------------------ 0

Viperin-Homo sapiens --------------------------------MWV------------------------- 3

Viperin-Candidatus ------------------------------------------------------------ 0

Viperin-Chlamydomonas ------------------------------------------------------------ 0

MoaA-Xanthomonas ------------------------------------------------------------ 0

MoaA-Volvox carteri SSSSSALQA-TAAAATARDRARAALRNVPAFTPARIGGCSSNSSNPSGPPANGCGGAAAA 293

MoaA-Dunaliella HAHFSTAAPV-PKPRRQ---TRLRIEDVPMFQIERKDSSKRDSSN--------------- 138

MOCS1A-Homo sapiens -------------------------------------------AG--------------- 18

MoaA-Physcomitrium FHGYSGVSAVVCKQGSVVSFHALALEKEPQPEDERRDGEVDRHSN--------------- 73

MoaA-Zostra marina ----------MAMRRRVMD--LLRRESVFR----ITP----------------------- 21

CNX2-Nicotiana ----------MASESQVFQ--LFNLQASFS----SRASLLSASSS--------------- 29

Viperin-Shewanella ------------------------------------------------------------ 0

Viperin-Fibrobacter ------------------------------------------------------------ 0

Viperin-Homo sapiens ------------------------------LTPAAFAGKLLS------------------ 15

Viperin-Candidatus ------------------------------------------------------------ 0

V Viperin-Chlamydomonas ---------------------------------------MLT------------------ 3

MoaA-Xanthomonas ----------------------------------------------------MG-ALLLP 7

MoaA-Volvox carteri AALPRSEVSAALPQPVPAAPVLPAPPAPPHPASPTPVDWRG-----LLQRARQV-LGSRQ 347

MoaA-Dunaliella -----------------RDPKLQPSPS------HAGLDWRE---MLARARASQE-AESTV 171

MOCS1A-Homo sapiens -----------------GSPCASSQPGSRGPCFLPGLSSQE------VSRRRQF-LREHA 54

MoaA-Physcomitrium -----------------EKPRS---DNLREKSSIEGVSPVRSSTIEADPRYQRR-HAEEA 112

MoaA-Zostra marina -------------------------------NYLVGACSKA--ISQSTPATIC---EETN 45

CNX2-Nicotiana -----------------G--------QNTTTSQLNGSSSKM--YSTSC-ETLSE-DSPKD 60

Viperin-Shewanella ------------------------------------------------------------ 0

Viperin-Fibrobacter ------------------------------------------------------------ 0

Viperin-Homo sapiens ----------VFRQPL---SSLW-RSLVPLFCWLRATFWLL-----ATKRRKQQLVLRGP 56

Viperin-Candidatus ------------------------------------------------------------ 0

Viperin-Chlamydomonas ----------SFQAGS-----------GSLACQAP--------------R-----RLPSP 23

MoaA-Xanthomonas NLATAPMQDRYGR-----PLRDLRLSVIEACNFRCGYCMPADRVPDDYG--FDSRQR-LS 59

MoaA-Volvox carteri VAGAEMLTDKFSR-----IHTYLRISLTERCNLRCTYCMPDEGVALTP-----TPQL-LT 396

MoaA-Dunaliella QGGPAMVTDTFSR-----VHTYLRISLTEKCNLRCQYCMPAEGIALTP-----NQRL-LT 220

MOCS1A-Homo sapiens APFSAFLTDSFGR-----QHSYLRISLTEKCNLRCQYCMPEEGVPLTP-----KANL-LT 103

MoaA-Physcomitrium DRVSDMLTDSHGR-----RHNYLRISLTERCNLRCHYCMPAEGVELTP-----NSGL-LS 161

MoaA-Zostra marina TSKSDMLVDSFGR-----QHTYLRISLTERCNLRCHYCMPSDGVDLTP-----NSSL-LS 94

CNX2-Nicotiana KPISDMLIDSFGR-----LHTYLRISLTERCNLRCHYCMPAEGVELTP-----SAQL-LS 109

Viperin-Shewanella --------------MSKANQLVINYHITEKCNYDCHYCYAKWAKPNELHRNLDDMKLVLS 46

Viperin-Fibrobacter ---------------MNIKTIVINWHITEACNYRCSFCFAKWNKPAEIWSNPENVRKIIV 45

Viperin-Homo sapiens DET----KEEEEDPPLPTTPTSVNYHFTRQCNYKCGFCFHTAKTSFVLP----------- 101

Viperin-Candidatus ---MQAIFSGADKQQNNALPAAVNWHFWPWCNYACKFCFASFEDIPRGDRLG-------- 49

Viperin-Chlamydomonas PQTAAVLHQQYCRSAQLALPPTVNWHLEPRCNYHCKFCFATFSDIPSSEVVK-------- 75

:. . ** * :*

MoaA-Xanthomonas --FDQLETLVRA--FVSVGVTKVRLTGGEPLLRRDLPSLIARLTAIE---GIEDLALTTN 112

MoaA-Volvox --SREIMRLARI--FVEAGVTKIRLTGGEPTLRRDITDLIRQLSSLR-PMGLRSVAITSN 451

MoaA-Dunaliella --TQEIMRLTRL--FVEAGINKVRLTGGEPTLRPDLVDLCHQLKVLP---GLETIAITTN 273

MOCS1A-Homo sapiens --TEEILTLARL--FVKEGIDKIRLTGGEPLIRPDVVDIVAQLQRLE---GLRTIGVTTN 156

MoaA-Physcomitrium --QEEIIRIAST--FVAGGVDKIRLTGGEPSIRSDIEEICEQLRSLP---GLQNLAMTSN 214

MoaA-Zostra marina --SDEIIRVANL--FVSSGVDKIRLTGGEPTIRKDIEDICSRLSNME---GLKSLGITTN 147

CNX2-Nicotiana --QDEIVRLASL--FVSSGVNKIRLTGGEPTIRKDIEELCLQLSSLK---GLKTLAMTTN 162

Viperin-Shewanella RLADYFLSPNPIQQQLQYQSVRLNFAGGEPLLLKQ--LFIEALDYAIE--LGFKTSIITN 102

Viperin-Fibrobacter NIRDHFRSQG-------VFNIRLNIVGGEPIMFPE--RLWNVVETAYE--NGMDISIITN 94

Viperin-Homo sapiens --LEEAKRGLLLLK--EAGMEKINFSGGEPFLQDRGEYLGKLVRFCKVELRLPSVSIVSN 157

Viperin-Candidatus -KEE-ALKIPAMLA--AAGAEKITFVGGEPTLCP---YLGDLVIAAKK--ADLVTCIVSN 100

Viperin-Chlamydomonas -DADLLLAVPPLLA--AAGVSKITFVGGEPLLHP---LLPELLAAAKG--AGLVTSLVSN 127

:: : **** : : : : :*

MoaA-Xanthomonas GTLLARQAVALRQAGLRRITVSMDALEPALFRRMNGDRGEIARVL--------------- 157

MoaA-Volvox GIVLARQLPELKEAGLTAVNISLDTLRGERFEQLARRPG-HKRVL--------------- 495

MoaA-Dunaliella GITLSRNLPALQEAGLSAINISLDTLRPERFEVMSRRPG-HDKVM--------------- 317

MOCS1A-Homo sapiens GINLARLLPQLQKAGLSAINISLDTLVPAKFEFIVRRKG-FHKVM--------------- 200

MoaA-Physcomitrium GIILSRKLFRLQAAGLNQLNISLDTLVPAKFELLTRRKG-HNKVL--------------- 258

MoaA-Zostra marina GIVLARKLPKLREYGLNLLNISLDTLVPSKFEFMTRRKG-HNKVL--------------- 191

CNX2-Nicotiana GITLGKKLPKLKDSGLNLVNISLDTLVPAKFEFMTRRKG-HQRVM--------------- 206

Viperin-Shewanella GHLISDQFIIEHSHKLQLLGISYDSCHIGGQQKIGRITASGKVLSAARLQS--------- 153

Viperin-Fibrobacter GSHLENIR--PFAHLISQVGISIDSLDHETNMKIGRECG-GKTICLDALRQ--------- 142

Viperin-Homo sapiens GSLIRERWFQNYGEYLDILAISCDSFDEEVNVLIGRGQGKKNH----------------- 200

Viperin-Candidatus GSGLTEQFLSEYSPYIDWIGLSIDASNDDLHEQIGRGLKKDLAIQ--------------- 145

Viperin-Chlamydomonas GSLLTEDWLRRMQGHLDWIAFSVDASDDALHAALGRGTTRETGVTMPRHSSSSSSSTKNS 187

* : : : .* *: :

MoaA-Xanthomonas --------------------------------------------------AGIAAAEQAG 167

MoaA-Volvox --------------------------------------------------ESIRTAVGLG 505

MoaA-Dunaliella --------------------------------------------------RSIDKALSMG 327

MOCS1A-Homo sapiens --------------------------------------------------EGIHKAIELG 210

MoaA-Physcomitrium --------------------------------------------------QSIDTALGLG 268

MoaA-Zostra marina --------------------------------------------------EAIDVAIDLG 201

CNX2-Nicotiana --------------------------------------------------ESIDAAVELG 216

Viperin-Shewanella -----------------------------------------------IFHQV--KRQSPT 164

Viperin-Fibrobacter -----------------------------------------------KIEDL--RKVNPD 153

Viperin-Homo sapiens ---------------------------------------------VENLQKLRRWCRDYR 215

Viperin-Candidatus -----------------------------------------RSHHLELSKIVWGRCQSFG 164

Viperin-Chlamydomonas SHRKPSGDGSSRGGATGSGTATSPAVGRGGGGGDVGGGVGTRGGHLARVERLWGVAQGLG 247

MoaA-Xanthomonas FQRLKINCVVQRG---VNEDQVLPLVEHFRGTGHVLRFIEFMDVGSCNGWTPDAVVTSAQ 224

MoaA-Volvox YDPVKVNVVVMRG---VNDDEVADFAALTRDQPINVRFIEYMPFD-GNVWSDSKMVPYRE 561

MoaA-Dunaliella YNPVKLNVVVMRG---VNDDELNDFVALTRDHPINVRFIEYMPFD-GNVWSDKKMVPYRE 383

MOCS1A-Homo sapiens YNPVKVNCVVMRG---LNEDELLDFAALTEGLPLDVRFIEYMPFD-GNKWNFKKMVSYKE 266

MoaA-Physcomitrium FSPVKVNTVVMRG---LNDDEILDFVEITRDRDINVRFIEFMPFD-GNVWNPKKLVSYVE 324

MoaA-Zostra marina YNPVKVNCVIMRG---INDDEICNFVELTKEKPINVRFIEFMPFD-GNVWNVKKLVSYAE 257

CNX2-Nicotiana YNPVKVNCVVMRG---FNDDEICDFVELTRERPINVRFIEFMPFD-GNVWNVKKLVPYAE 272

Viperin-Shewanella -TELKINTVVNQF--NVEEDFTALITAL---QPNKWKVLRVLPVF-DSIQTIR------- 210

Viperin-Fibrobacter -IKIKLNTVVSKH--NFNEVLVERFAEL---HIDKWKILRQRPFN-GN-SGIS------- 198

Viperin-Homo sapiens -VAFKINSVINRF--NVEEDMTEQIKAL---NPVRWKVFQCLLIE-GENCGEDALREAER 268

Viperin-Candidatus -IRMKLNTVVCSV--NKDDTMLELVRQL---RPGRWKIFEVLPVA-GQND-EF----IEG 212

Viperin-Chlamydomonas -YRLKLNTVVTAPCLGDVGGMVELVGRL---RPERWKVFQVLPIT-GQTQPEH----IAP 298

.*:* *: . :.:. . .

MoaA-Xanthomonas LH--ERIHARWPLV---------------------------------------------- 236

MoaA-Volvox LI--SRIQAAFPYT--------------------------------------------PL 575

MoaA-Dunaliella MM--ARVREALQQQHQLDQRQQQQQQQQQELSPVETAASNSHQSSSSSSSSSCSEHNQLL 441

MOCS1A-Homo sapiens ML--DTVRQQWPE----------------------------------------------L 278

MoaA-Physcomitrium MM--DTIKNKFPS----------------------------------------------I 336

MoaA-Zostra marina MF--DIVTKRFEG----------------------------------------------V 269

CNX2-Nicotiana ML--DKVGKQFTG----------------------------------------------L 284

Viperin-Shewanella ----DPQFEAFV------------------------------------------------ 218

Viperin-Fibrobacter ----DYQFYAFLRNNYNEGLMQ-------------------------------------- 216

Viperin-Homo sapiens FVIGDEEFERFLERHK-------------------------------------------- 284

Viperin-Candidatus LVLKDGEFDTWLSRHK-------------------------------------------- 228

Viperin-Chlamydomonas LLTTEAQFADWVAAAS-------------------------------------------- 314

MoaA-Xanthomonas ALDAHYTGEVAQRHAFADGAG------EVGF---VSSVSVPFCGDCQRARVSADGHLYTC 287

MoaA-Volvox ERLDDPAGEVAKNFRLWGHRG------SVSF---ITSMTQHFCSDCNRLRLLADGNLKVC 626

MoaA-Dunaliella ERLADPNGEVAKNFRVPGHAG------IISF---VTSMTSHFCGECNRLRLMADGNLKVC 492

MOCS1A-Homo sapiens EKVPEEESSTAKAFKIPGFQG------QISF---ITSMSEHFCGTCNRLRITADGNLKVC 329

MoaA-Physcomitrium YRLKDHPTDTAKNFRVEGYLG------TVSF---ITSMTQHFCSGCNRLRLMADGNLKVC 387

MoaA-Zostra marina KRCQDHPSDTAKNFSIDGHRG------TISF---ITSMTEHFCGGCNRLRLLADGNFKVC 320

CNX2-Nicotiana QRIQDHPTETAKNFRIDGHQG------SVSF---ITSMTEHFCAGCNRLRLLADGNFKVC 335

Viperin-Shewanella --------------------ARHQAVKQVMSVENNDSMTNS------YLMLSPDGAFFQN 252

Viperin-Fibrobacter ANVLKRHTELPLSFLIDGSDRQDQETKQVIYIEDKDVMTES------YLMISPDGRLFQN 270

Viperin-Homo sapiens --------------------EV-----SCLVPESNQKMKDS------YLILDEYMRFLNC 313

Viperin-Candidatus --------------------D-VELDGIQFVPESNDLMRGS------YAMLDALGRFYSN 261

Viperin-Chlamydomonas --------------------VVQELYGVPLVPESNSHMHGS------YAMLDARGRFYQD 348

: : :

MoaA-Xanthomonas LFASQGHDLKPALANGEPALATHLWQCWSVRGDRYSEVRASVPRRGKP------------ 335

MoaA-Volvox LFGAAEVSLRDAMRGGASDDDLRAIITAAVGRKRAAHAGMFELAASAN------------ 674

MoaA-Dunaliella LFGASEVSLRDAMRGGATDEDLRLIIGAAVRRKKAKHAGMFEIAATQN------------ 540

MOCS1A-Homo sapiens LFGNSEVSLRDHLRAGASEQELLRIIGAAVGRKKRQHAGMFSISQMKN------------ 377

MoaA-Physcomitrium LFGPSEVSLRDAVRSGMQESELQQVISDAVKRKKAAHAGMFELARTQN------------ 435

MoaA-Zostra marina LFGPSEVSLRDPIRSGTDDDGLRDIIGAAVKRKKASHAGMFDIAKTAN------------ 368

CNX2-Nicotiana LFGPSEVSLRDPLRLGEGDDKLREIIGAAVKRKKASHAGMFDIAKTPN------------ 383

Viperin-Shewanella GDQAQGYFKSRPLLTTPIDVALAETGFDAVKFAQRYVSATQVLGAA-------------- 298

Viperin-Fibrobacter GSDE--YTYSRPLTEVPFAEALSDIRFDSEKFESRYATWPTQEAVYEMEYFFHLVEDDYD 328

Viperin-Homo sapiens RKGR-KD-PSKSILDVGVEEAIKF------------------------------------ 335

Viperin-Candidatus VDGR-HQYASP-ILDVGVEEGWNE------------------------------------ 283

Viperin-Chlamydomonas VNGE-GYEYGPSIFD-----CWQGRLLESLALDSHAHPHQPRLAAAPSH--DAAVGTSAD 400

:

MoaA-Xanthomonas ------------------------------------------------------------ 335

MoaA-Volvox ------------------------------------------------------------ 674

MoaA-Dunaliella ------------------------------------------------------------ 540

MOCS1A-Homo sapiens ------------------------------------------------------------ 377

MoaA-Physcomitrium ------------------------------------------------------------ 435

MoaA-Zostra marina ------------------------------------------------------------ 368

CNX2-Nicotiana ------------------------------------------------------------ 383

Viperin-Shewanella ------------------------------------------------------------ 298

Viperin-Fibrobacter DFDC--FTDLSDD----------------------------------------------- 339

Viperin-Homo sapiens ------------------------------------------------------------ 335

Viperin-Candidatus ------------------------------------------------------------ 283

Viperin-Chlamydomonas DVHAAAMAVLMARPTSSPAAVGSGGVARGAAAGDGDGDGGNSNGAGPNARTQMQMEAGRE 460

MoaA-Xanthomonas ---------------VEMFLIGG---------------------------------- 343

MoaA-Volvox ---------------RPMITIGG---------------------------------- 682

MoaA-Dunaliella ---------------RPMITIGG---------------------------------- 548

MOCS1A-Homo sapiens ---------------RPMILIGG---------------------------------- 385

MoaA-Physcomitrium ---------------RPMIHIGG---------------------------------- 443

MoaA-Zostra marina ---------------RPMIHIGG---------------------------------- 376

CNX2-Nicotiana ---------------RPMIHIGGGSSYPYDVPDYAYPYDVPDYAYPYDVPDYA---- 421

Viperin-Shewanella --------------------------------------------------------- 298

Viperin-Fibrobacter --------------------------------------------------------- 339

Viperin-Homo sapiens -----------SGFDEKMFL-KRGGKYIWSKADLKLDW------------------- 361

Viperin-Candidatus -----------TRFLEERFI-ERGGIYDW---------------------------- 300

Viperin-Chlamydomonas AVMDAWGAVAGPAFRAEAFA-TRGGVYEWGQGGEALEEQQRAWGEAASSSGGAESDI 516

**Supplementary Figure 1. Protein alignment of Viperin and Viperin-like proteins from different species**. Alignment of amino acid sequences from Nicotiana sylvestris (XP_009771542.), Homo sapiens (NP_542388.2), Homo sapiens (NP_005934.2), Xanthomonas oryzae ( WP_011257906.1), Shewanella sp. cp20 (WP_052813773)), Fibrobacter sp. UWT3 (WP_097037079.1), Candidatus Poseidoniaceae archaeon (MDB2540369.1), Chlamydomonas reinhardtii (XP_042926382.1), Volvox carteri f. nagariensis (Vocar.0006s0432.1), Physcomitrium patens (XP_024378973.1), Zostera marina (KMZ71380.1), Dunaliella salina (KAF5832982.1). The radical SAM domain is labelled in yellow. Alignment was generated using the CLUSTAL (1.2.4) multiple sequence alignment tool.


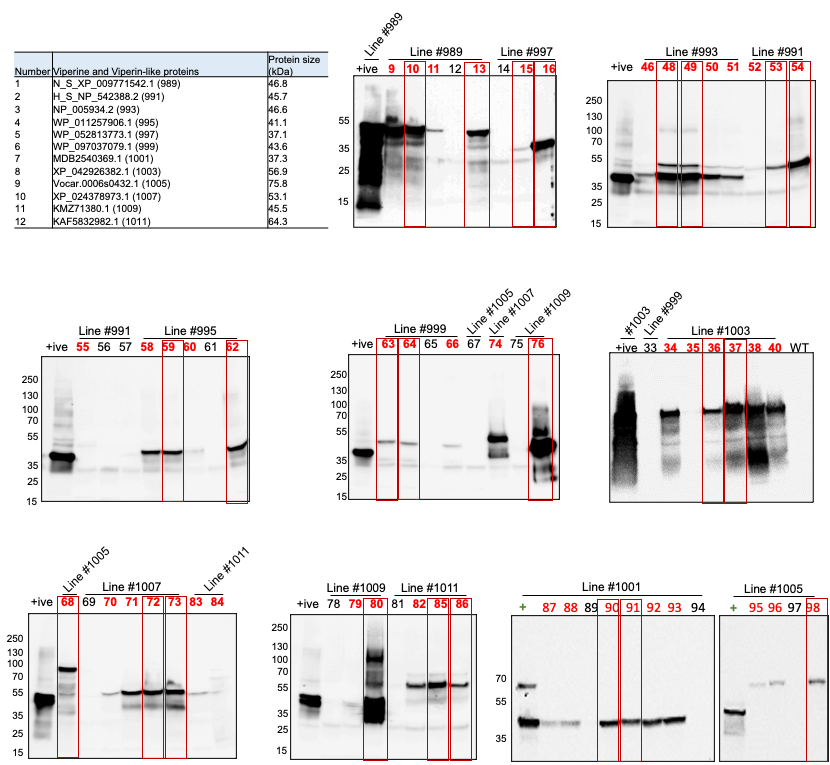


**Supplementary Figure 2: Protein expression confirmation in N.bentamiana lines overexpression lines.**

Western blot confirmation of *N.benthamiana* lines overexpressing our proteins of interest. For protein confirmation, total proteins from leaves were extracted, and a western blot was performed with an HA antibody. The samples labelled in red indicate positive plant lines. The samples labelled in the box were the ones used in viral interference assays. The labelling of proteins is listed in the figure, along with the size of each protein in kilo Dalton (kDa).


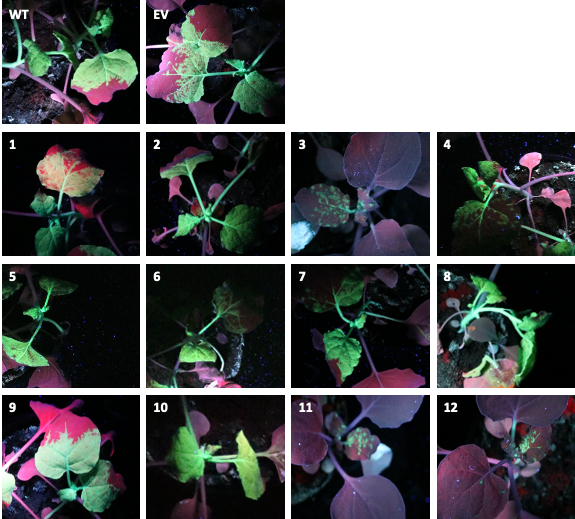


**Supplementary Figure 3: Screening antiviral activity of identified proteins against TuMV-GFP**

N. benthamiana plants with stable overexpression of various antiviral proteins were agroinfiltrated with TuMV-GFP using agrobacteriu. The systemic leaves were observed for the presence of GFP fluorescence signals 7 days post-agroinfiltration (dai) using a handheld UV device, and photographs were captured with a digital camera. EV represents empty vector.


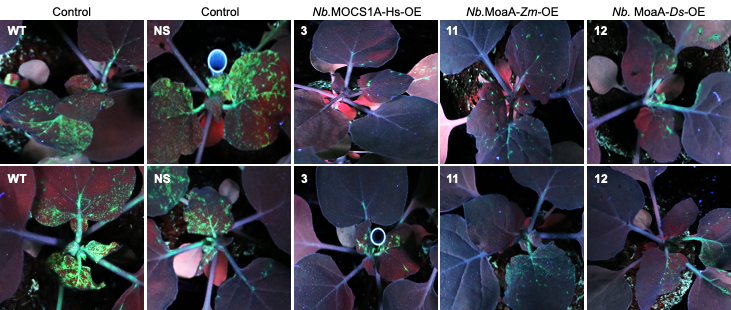
**Supplementary Figure 4: Confirmation of TuMV resistant plants with Sap inoculation**

N. *benthamiana* plants overexpressing different antiviral proteins against TuMV were inoculated with the TuMV-GFP virus via sap application. To assess virus spread, GFP fluorescence in the systemic leaves was assessed 7 days after inoculation, using a handheld UV light. Photographs of the fluorescence were capture with a digital camera. NS represent plants expressing non-specific protein.

**Supplementary Figure 5: Screening antiviral activity of identified proteins against PVX-GFP**

N. benthamiana plants with stable overexpression of various antiviral proteins were agroinfiltrated with PVX-GFP via agrobacterium. The systemic leaves were observed for the presence of GFP fluorescence signals 7 days post-agroinfiltration (dai) using a handheld UV device, and photographs were captured with a digital camera. EV represents empty vector.


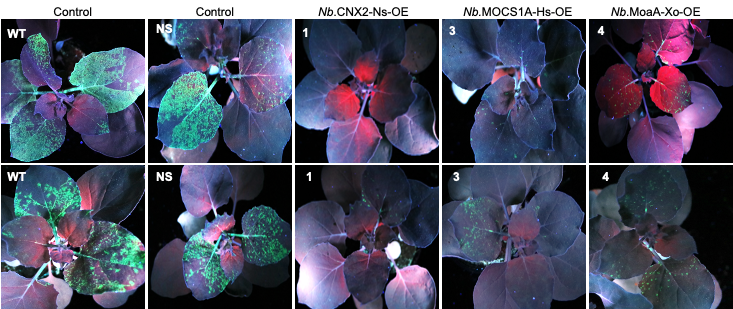


**Supplementary Figure 6: Confirmation of PVX resistant plants with Sap inoculation**

N.*benthamiana* plants overexpressing antiviral proteins against PVX were sap inoculated with a PVX-GFP virus. Seven days after inoculation, the systemic leaves were examined for GFP fluorescence using a handheld UV light. Images of the fluorescence were taken with a digital camera. NS represent plants expressing non-specific protein.

**Table 1. Sequences of proteins used in this study**

A linker and 3xHA-tag sequences (including the stop codon) are added to the C-terminus of each protein sequence.

Linker: GSS

3xHA: YPYDVPDYAYPYDVPDYAYPYDVPDYA

| **Sequence ID** | **Source** | **Protein Sequence** | **Coding sequence** |
| --- | --- | --- | --- |
| >N_S(XP_009771542.1) | [*Nicotiana sylvestris*](https://www.ncbi.nlm.nih.gov/Taxonomy/Browser/wwwtax.cgi?id=4096) | MASESQVFQLFNLQASFSSRASLLSASSSGQNTTTSQLNGSSSKMYSTSCETLSEDSPKDKPISDMLIDSFGRLHTYLRISLTERCNLRCHYCMPAEGVELTPSAQLLSQDEIVRLASLFVSSGVNKIRLTGGEPTIRKDIEELCLQLSSLKGLKTLAMTTNGITLGKKLPKLKDSGLNLVNISLDTLVPAKFEFMTRRKGHQRVMESIDAAVELGYNPVKVNCVVMRGFNDDEICDFVELTRERPINVRFIEFMPFDGNVWNVKKLVPYAEMLDKVGKQFTGLQRIQDHPTETAKNFRIDGHQGSVSFITSMTEHFCAGCNRLRLLADGNFKVCLFGPSEVSLRDPLRLGEGDDKLREIIGAAVKRKKASHAGMFDIAKTPNRPMIHIGG**GSSYPYDVPDYAYPYDVPDYAYPYDVPDYA*** | ATGGCCTCTGAGAGCCAGGTGTTCCAGCTGTTTAACCTGCAGGCCTCTTTTAGCTCCAGGGCCTCTCTGCTGAGCGCCTCTAGCTCCGGCCAGAACACCACAACCAGCCAGCTGAATGGCTCTAGCTCCAAGATGTACTCCACATCTTGCGAGACCCTGTCCGAGGACTCTCCAAAGGATAAGCCCATCTCCGACATGCTGATCGATTCTTTTGGCAGGCTGCACACATACCTGCGCATCTCCCTGACCGAGAGGTGTAATCTGCGCTGCCACTATTGTATGCCTGCCGAGGGCGTGGAGCTGACACCATCTGCCCAGCTGCTGAGCCAGGACGAGATCGTGCGGCTGGCCTCTCTGTTCGTGTCTAGCGGCGTGAACAAGATCCGGCTGACAGGCGGCGAGCCAACCATCAGAAAGGACATCGAGGAGCTGTGCCTGCAGCTGTCCTCTCTGAAGGGCCTGAAGACACTGGCCATGACAACCAATGGCATCACCCTGGGCAAGAAGCTGCCTAAGCTGAAGGACAGCGGCCTGAACCTGGTGAATATCTCCCTGGATACACTGGTGCCAGCCAAGTTCGAGTTTATGACCCGGAGAAAGGGCCACCAGAGAGTGATGGAGAGCATCGACGCAGCAGTGGAGCTGGGATACAACCCTGTGAAAGTGAATTGCGTGGTCATGAGGGGCTTTAACGACGATGAGATTTGCGATTTCGTGGAGCTGACAAGGGAGCGCCCAATCAATGTGCGCTTCATCGAGTTTATGCCCTTCGACGGCAACGTGTGGAATGTGAAGAAGCTGGTGCCCTATGCCGAGATGCTGGATAAAGTGGGCAAGCAGTTTACCGGCCTGCAGAGAATCCAGGACCACCCTACAGAGACCGCCAAGAACTTCAGAATCGATGGCCACCAGGGCAGCGTGTCCTTTATCACAAGCATGACCGAGCACTTCTGCGCCGGCTGTAACCGGCTGAGACTGCTGGCCGACGGCAACTTCAAGGTGTGCCTGTTCGGACCAAGCGAGGTGTCCCTGCGGGACCCCCTGAGACTGGGAGAGGGCGACGATAAGCTGAGGGAGATCATCGGAGCAGCAGTGAAGCGCAAGAAGGCCTCCCACGCCGGCATGTTCGACATCGCCAAGACCCCCAATCGGCCTATGATCCACATCGGCGGCGGCAGCTCCTACCCTTATGACGTGCCAGATTACGCCTATCCCTACGACGTGCCTGATTATGCCTACCCCTACGACGTGCCCGATTATGCCTGA |
| >H_S (NP_542388.2) | [*Homo sapiens*](https://www.ncbi.nlm.nih.gov/Taxonomy/Browser/wwwtax.cgi?id=9606) | MWVLTPAAFAGKLLSVFRQPLSSLWRSLVPLFCWLRATFWLLATKRRKQQLVLRGPDETKEEEEDPPLPTTPTSVNYHFTRQCNYKCGFCFHTAKTSFVLPLEEAKRGLLLLKEAGMEKINFSGGEPFLQDRGEYLGKLVRFCKVELRLPSVSIVSNGSLIRERWFQNYGEYLDILAISCDSFDEEVNVLIGRGQGKKNHVENLQKLRRWCRDYRVAFKINSVINRFNVEEDMTEQIKALNPVRWKVFQCLLIEGENCGEDALREAERFVIGDEEFERFLERHKEVSCLVPESNQKMKDSYLILDEYMRFLNCRKGRKDPSKSILDVGVEEAIKFSGFDEKMFLKRGGKYIWSKADLKLDW**GSSYPYDVPDYAYPYDVPDYAYPYDVPDYA*** | ATGTGGGTGCTGACCCCAGCAGCCTTCGCCGGCAAGCTGCTGAGCGTGTTCCGGCAGCCTCTGAGCAGCCTGTGGAGAAGCCTGGTGCCACTGTTCTGCTGGCTGAGGGCCACCTTTTGGCTGCTGGCCACAAAGCGGAGAAAGCAGCAGCTGGTGCTGAGGGGACCTGACGAGACAAAGGAGGAGGAGGAGGACCCCCCTCTGCCAACCACACCCACCAGCGTGAACTACCACTTCACAAGGCAGTGTAATTATAAGTGCGGCTTCTGTTTTCACACCGCCAAGACATCCTTTGTGCTGCCACTGGAGGAGGCCAAGAGGGGACTGCTGCTGCTGAAGGAGGCAGGCATGGAGAAGATCAACTTCAGCGGAGGAGAGCCCTTTCTGCAGGACAGGGGCGAGTACCTGGGCAAGCTGGTGAGGTTCTGCAAGGTGGAGCTGCGCCTGCCTTCCGTGTCTATCGTGTCTAACGGCAGCCTGATCCGGGAGAGATGGTTCCAGAATTACGGCGAGTACCTGGACATCCTGGCCATCTCCTGCGACTCTTTTGATGAGGAGGTGAACGTGCTGATCGGAAGGGGACAGGGCAAGAAGAACCACGTGGAGAATCTGCAGAAGCTGAGGCGCTGGTGTAGGGACTACCGCGTGGCCTTCAAGATCAATTCTGTGATCAACCGGTTTAATGTGGAGGAGGATATGACCGAGCAGATCAAGGCCCTGAACCCTGTGAGATGGAAGGTGTTCCAGTGCCTGCTGATCGAGGGCGAGAATTGTGGAGAGGACGCACTGAGGGAGGCAGAGCGGTTCGTGATCGGCGATGAGGAGTTCGAGCGGTTTCTGGAGAGACACAAGGAGGTGAGCTGCCTGGTGCCAGAGTCCAACCAGAAGATGAAGGACTCCTACCTGATCCTGGATGAGTATATGCGGTTCCTGAATTGTCGGAAGGGCAGAAAGGACCCCAGCAAGTCCATCCTGGATGTGGGCGTGGAGGAGGCCATCAAGTTCTCCGGCTTTGACGAGAAGATGTTTCTGAAGAGAGGCGGCAAGTACATCTGGTCTAAGGCCGACCTGAAGCTGGATTGGGGCTCTAGCTACCCTTATGACGTGCCAGATTACGCCTATCCCTACGACGTGCCTGATTATGCCTACCCCTACGACGTGCCCGATTATGCCTGA |
| >NP_005934.2 | [*Homo sapiens*](https://www.ncbi.nlm.nih.gov/Taxonomy/Browser/wwwtax.cgi?id=9606) | MWKSWKLRTDVRVREGAGGSPCASSQPGSRGPCFLPGLSSQEVSRRRQFLREHAAPFSAFLTDSFGRQHSYLRISLTEKCNLRCQYCMPEEGVPLTPKANLLTTEEILTLARLFVKEGIDKIRLTGGEPLIRPDVVDIVAQLQRLEGLRTIGVTTNGINLARLLPQLQKAGLSAINISLDTLVPAKFEFIVRRKGFHKVMEGIHKAIELGYNPVKVNCVVMRGLNEDELLDFAALTEGLPLDVRFIEYMPFDGNKWNFKKMVSYKEMLDTVRQQWPELEKVPEEESSTAKAFKIPGFQGQISFITSMSEHFCGTCNRLRITADGNLKVCLFGNSEVSLRDHLRAGASEQELLRIIGAAVGRKKRQHAGMFSISQMKNRPMILIGG**GSSYPYDVPDYAYPYDVPDYAYPYDVPDYA*** | ATGTGGAAGAGCTGGAAGCTGAGGACCGACGTGAGAGTGAGGGAGGGAGCAGGAGGCTCTCCATGCGCCAGCTCCCAGCCTGGCAGCCGCGGCCCATGTTTCCTGCCAGGACTGTCTAGCCAGGAGGTGTCCCGGAGAAGGCAGTTTCTGAGGGAGCACGCAGCACCATTCAGCGCCTTTCTGACCGATTCCTTCGGCCGCCAGCACTCTTACCTGCGGATCAGCCTGACAGAGAAGTGCAACCTGAGATGCCAGTATTGTATGCCAGAGGAGGGCGTGCCCCTGACCCCTAAGGCCAATCTGCTGACCACAGAGGAGATCCTGACACTGGCCAGGCTGTTTGTGAAGGAGGGCATCGACAAGATCAGACTGACCGGAGGAGAGCCTCTGATCAGGCCAGACGTGGTGGACATCGTGGCCCAGCTGCAGAGACTGGAGGGCCTGAGGACAATCGGCGTGACCACAAACGGCATCAATCTGGCAAGGCTGCTGCCACAGCTGCAGAAGGCAGGACTGTCCGCCATCAACATCTCTCTGGATACCCTGGTGCCTGCCAAGTTCGAGTTTATCGTGCGCCGGAAGGGCTTCCACAAAGTGATGGAGGGCATCCACAAGGCCATCGAGCTGGGCTACAACCCTGTGAAAGTGAATTGCGTGGTCATGAGAGGCCTGAATGAGGACGAGCTGCTGGATTTCGCCGCCCTGACCGAGGGACTGCCTCTGGACGTGAGGTTCATCGAGTACATGCCATTTGATGGCAACAAGTGGAACTTCAAGAAGATGGTGAGCTATAAGGAGATGCTGGACACAGTGCGGCAGCAGTGGCCAGAGCTGGAGAAGGTGCCCGAGGAGGAGTCCTCTACCGCCAAGGCCTTCAAGATCCCAGGCTTTCAGGGCCAGATCAGCTTCATCACATCTATGAGCGAGCACTTTTGCGGCACCTGTAACCGCCTGCGGATCACAGCCGACGGCAACCTGAAGGTGTGCCTGTTCGGCAATAGCGAGGTGTCCCTGAGGGATCACCTGAGGGCAGGAGCCAGCGAGCAGGAGCTGCTGAGGATCATCGGAGCAGCAGTGGGCAGAAAGAAGAGGCAGCACGCCGGCATGTTTTCCATCTCTCAGATGAAGAATAGGCCAATGATCCTGATCGGAGGAGGCAGCTCCTACCCCTATGACGTGCCTGATTACGCCTATCCATACGACGTGCCCGATTATGCCTACCCTTATGACGTGCCAGATTATGCCTGA |
| >WP_011257906.1 | [*Xanthomonas oryzae*](https://www.ncbi.nlm.nih.gov/Taxonomy/Browser/wwwtax.cgi?id=347) | MGALLLPNLATAPMQDRYGRPLRDLRLSVIEACNFRCGYCMPADRVPDDYGFDSRQRLSFDQLETLVRAFVSVGVTKVRLTGGEPLLRRDLPSLIARLTAIEGIEDLALTTNGTLLARQAVALRQAGLRRITVSMDALEPALFRRMNGDRGEIARVLAGIAAAEQAGFQRLKINCVVQRGVNEDQVLPLVEHFRGTGHVLRFIEFMDVGSCNGWTPDAVVTSAQLHERIHARWPLVALDAHYTGEVAQRHAFADGAGEVGFVSSVSVPFCGDCQRARVSADGHLYTCLFASQGHDLKPALANGEPALATHLWQCWSVRGDRYSEVRASVPRRGKPVEMFLIGG**GSSYPYDVPDYAYPYDVPDYAYPYDVPDYA*** | ATGGGCGCCCTGCTGCTGCCTAACCTGGCAACCGCACCAATGCAGGACAGATACGGCAGACCACTGAGGGATCTGCGCCTGTCCGTGATCGAGGCCTGCAACTTCCGGTGCGGATACTGTATGCCAGCAGACCGCGTGCCTGACGATTATGGCTTCGACTCCCGGCAGAGACTGTCTTTTGATCAGCTGGAGACACTGGTGAGGGCCTTCGTGAGCGTGGGAGTGACCAAGGTGCGCCTGACAGGAGGAGAGCCACTGCTGCGGAGAGACCTGCCTTCCCTGATCGCAAGGCTGACCGCAATCGAGGGAATCGAGGATCTGGCCCTGACCACAAACGGCACACTGCTGGCCAGGCAGGCAGTGGCCCTGAGGCAGGCAGGACTGAGGCGCATCACCGTGTCTATGGACGCCCTGGAGCCCGCCCTGTTCCGGAGAATGAATGGCGATAGGGGAGAGATCGCCAGAGTGCTGGCCGGAATCGCTGCCGCCGAGCAGGCAGGATTTCAGCGGCTGAAGATCAACTGCGTGGTGCAGAGAGGCGTGAATGAGGATCAGGTGCTGCCTCTGGTGGAGCACTTCCGGGGCACCGGACACGTGCTGAGATTCATCGAGTTTATGGACGTGGGCTCTTGTAACGGCTGGACCCCTGATGCCGTGGTGACAAGCGCCCAGCTGCACGAGAGAATCCACGCAAGGTGGCCACTGGTGGCCCTGGACGCACACTACACAGGAGAGGTGGCACAGAGGCACGCCTTTGCCGATGGAGCAGGAGAAGTGGGATTCGTGAGCAGCGTGAGCGTGCCCTTTTGCGGCGACTGTCAGAGGGCCCGCGTGTCCGCCGATGGACACCTGTACACCTGCCTGTTCGCCTCTCAGGGACACGACCTGAAGCCAGCCCTGGCCAATGGAGAGCCCGCCCTGGCCACACACCTGTGGCAGTGTTGGAGCGTGCGGGGCGATAGATATAGCGAGGTGAGAGCCTCCGTGCCAAGGAGGGGCAAGCCAGTGGAGATGTTTCTGATCGGCGGCGGCTCTAGCTACCCCTATGACGTGCCTGATTACGCCTATCCATACGACGTGCCCGATTATGCCTACCCTTATGACGTGCCAGATTATGCCTGA |
| >WP_052813773.1 | [*Shewanella sp. cp20*](https://www.ncbi.nlm.nih.gov/Taxonomy/Browser/wwwtax.cgi?id=1521167) | MSKANQLVINYHITEKCNYDCHYCYAKWAKPNELHRNLDDMKLVLSRLADYFLSPNPIQQQLQYQSVRLNFAGGEPLLLKQLFIEALDYAIELGFKTSIITNGHLISDQFIIEHSHKLQLLGISYDSCHIGGQQKIGRITASGKVLSAARLQSIFHQVKRQSPTTELKINTVVNQFNVEEDFTALITALQPNKWKVLRVLPVFDSIQTIRDPQFEAFVARHQAVKQVMSVENNDSMTNSYLMLSPDGAFFQNGDQAQGYFKSRPLLTTPIDVALAETGFDAVKFAQRYVSATQVLGAA**GSSYPYDVPDYAYPYDVPDYAYPYDVPDYA*** | ATGTCTAAGGCCAACCAGCTGGTCATCAACTACCACATCACCGAGAAGTGCAACTATGACTGCCACTACTGTTATGCCAAGTGGGCCAAGCCAAACGAGCTGCACCGGAATCTGGACGATATGAAGCTGGTGCTGAGCCGGCTGGCCGATTACTTTCTGAGCCCCAACCCTATCCAGCAGCAGCTGCAGTATCAGAGCGTGAGACTGAATTTCGCAGGAGGAGAGCCACTGCTGCTGAAGCAGCTGTTTATCGAGGCCCTGGACTACGCCATCGAGCTGGGCTTCAAGACCTCCATCATCACAAATGGCCACCTGATCTCCGACCAGTTTATCATCGAGCACTCTCACAAGCTGCAGCTGCTGGGCATCTCTTATGATAGCTGTCACATCGGCGGCCAGCAGAAGATCGGAAGGATCACCGCCAGCGGCAAGGTGCTGTCCGCCGCAAGGCTGCAGAGCATCTTCCACCAGGTGAAGAGACAGTCCCCTACCACAGAGCTGAAGATCAACACAGTGGTGAACCAGTTCAATGTGGAGGAGGACTTTACCGCCCTGATCACAGCCCTGCAGCCTAATAAGTGGAAGGTGCTGCGGGTGCTGCCAGTGTTTGACTCCATCCAGACCATCCGGGACCCCCAGTTCGAGGCCTTTGTGGCAAGGCACCAGGCAGTGAAGCAGGTCATGTCTGTGGAGAACAATGATTCCATGACAAACTCTTACCTGATGCTGAGCCCTGACGGCGCCTTCTTTCAGAATGGCGATCAGGCCCAGGGCTATTTCAAGTCCAGGCCACTGCTGACCACACCAATCGACGTGGCCCTGGCCGAGACCGGATTCGATGCCGTGAAGTTTGCCCAGCGGTACGTGAGCGCCACACAGGTGCTGGGAGCAGCAGGCAGCTCCTACCCCTACGACGTGCCCGATTACGCCTATCCTTACGACGTGCCAGATTATGCCTACCCCTATGACGTGCCTGATTACGCCTGA |
| >WP_097037079.1 | [*Fibrobacter sp. UWT3*](https://www.ncbi.nlm.nih.gov/Taxonomy/Browser/wwwtax.cgi?id=1896225) | MNIKTIVINWHITEACNYRCSFCFAKWNKPAEIWSNPENVRKIIVNIRDHFRSQGVFNIRLNIVGGEPIMFPERLWNVVETAYENGMDISIITNGSHLENIRPFAHLISQVGISIDSLDHETNMKIGRECGGKTICLDALRQKIEDLRKVNPDIKIKLNTVVSKHNFNEVLVERFAELHIDKWKILRQRPFNGNSGISDYQFYAFLRNNYNEGLMQANVLKRHTELPLSFLIDGSDRQDQETKQVIYIEDKDVMTESYLMISPDGRLFQNGSDEYTYSRPLTEVPFAEALSDIRFDSEKFESRYATWPTQEAVYEMEYFFHLVEDDYDDFDCFTDLSDD**GSSYPYDVPDYAYPYDVPDYAYPYDVPDYA*** | ATGAACATCAAGACCATCGTGATCAATTGGCACATCACAGAGGCCTGCAACTACCGCTGCTCCTTCTGTTTTGCCAAGTGGAATAAGCCTGCCGAGATTTGGTCTAACCCAGAGAATGTGCGGAAGATCATCGTGAACATCAGGGATCACTTCCGCAGCCAGGGCGTGTTTAACATCAGGCTGAATATCGTGGGCGGCGAGCCCATCATGTTCCCTGAGCGCCTGTGGAACGTGGTGGAGACCGCCTATGAGAATGGCATGGACATCTCCATCATCACAAACGGCTCTCACCTGGAGAATATCAGACCTTTTGCCCACCTGATCTCTCAAGTGGGCATCTCTATCGACAGCCTGGATCACGAGACCAATATGAAGATCGGCAGGGAGTGCGGCGGCAAGACAATCTGTCTGGACGCCCTGCGGCAGAAGATCGAGGATCTGAGAAAGGTGAACCCAGACATCAAGATCAAGCTGAATACCGTGGTGTCCAAGCACAACTTCAATGAGGTGCTGGTGGAGAGATTTGCCGAGCTGCACATCGATAAGTGGAAGATCCTGCGGCAGAGACCCTTCAACGGCAATTCCGGCATCTCTGACTACCAGTTCTATGCCTTTCTGCGGAACAATTACAACGAGGGCCTGATGCAGGCCAATGTGCTGAAGAGACACACAGAGCTGCCCCTGAGCTTTCTGATCGACGGCTCCGATAGGCAGGACCAGGAGACCAAGCAGGTCATCTACATCGAGGACAAGGATGTGATGACAGAGAGCTATCTGATGATCTCCCCTGATGGCCGGCTGTTCCAGAACGGCTCTGACGAGTACACCTATAGCAGACCACTGACAGAGGTGCCCTTTGCCGAGGCCCTGAGCGACATCAGGTTCGACTCTGAGAAGTTTGAGAGCCGCTACGCAACCTGGCCAACACAGGAGGCCGTGTACGAGATGGAGTATTTCTTTCACCTGGTGGAGGACGATTATGACGATTTCGATTGTTTTACCGACCTGTCCGACGATGGCAGCTCCTACCCCTACGACGTGCCCGACTACGCCTATCCTTACGATGTGCCAGACTATGCCTACCCCTATGATGTGCCTGACTACGCCTGA |
| >MDB2540369.1 | [*Candidatus Poseidoniaceae archaeon*](https://www.ncbi.nlm.nih.gov/Taxonomy/Browser/wwwtax.cgi?id=2666346) | MQAIFSGADKQQNNALPAAVNWHFWPWCNYACKFCFASFEDIPRGDRLGKEEALKIPAMLAAAGAEKITFVGGEPTLCPYLGDLVIAAKKADLVTCIVSNGSGLTEQFLSEYSPYIDWIGLSIDASNDDLHEQIGRGLKKDLAIQRSHHLELSKIVWGRCQSFGIRMKLNTVVCSVNKDDTMLELVRQLRPGRWKIFEVLPVAGQNDEFIEGLVLKDGEFDTWLSRHKDVELDGIQFVPESNDLMRGSYAMLDALGRFYSNVDGRHQYASPILDVGVEEGWNETRFLEERFIERGGIYDW**GSSYPYDVPDYAYPYDVPDYAYPYDVPDYA*** | ATGCAGGCCATCTTCTCCGGCGCCGACAAGCAGCAGAACAATGCCCTGCCAGCCGCCGTGAACTGGCACTTCTGGCCCTGGTGCAATTACGCCTGCAAGTTCTGTTTTGCCTCTTTTGAGGACATCCCCCGGGGCGATAGACTGGGCAAGGAGGAGGCCCTGAAGATCCCTGCAATGCTGGCCGCCGCCGGAGCAGAGAAGATCACCTTCGTGGGAGGAGAGCCCACACTGTGCCCTTATCTGGGCGACCTGGTCATCGCCGCCAAGAAGGCCGATCTGGTGACCTGTATCGTGTCTAACGGCAGCGGCCTGACAGAGCAGTTTCTGTCCGAGTACTCTCCTTATATCGACTGGATCGGCCTGAGCATCGATGCCTCCAATGACGATCTGCACGAGCAGATCGGCCGCGGCCTGAAGAAGGATCTGGCCATCCAGCGGTCTCACCACCTGGAGCTGAGCAAGATCGTGTGGGGCCGCTGCCAGTCTTTCGGCATCCGGATGAAGCTGAACACCGTGGTGTGCAGCGTGAATAAGGACGATACAATGCTGGAGCTGGTGAGGCAGCTGAGGCCTGGCCGGTGGAAGATTTTCGAGGTGCTGCCAGTGGCCGGCCAGAACGACGAGTTCATCGAGGGCCTGGTGCTGAAGGACGGCGAGTTTGATACCTGGCTGAGCAGACACAAGGACGTGGAGCTGGATGGCATCCAGTTCGTGCCAGAGTCCAACGACCTGATGAGGGGCTCTTACGCCATGCTGGATGCCCTGGGCCGGTTCTACAGCAATGTGGACGGCAGGCACCAGTATGCCTCCCCCATCCTGGATGTGGGCGTGGAGGAGGGCTGGAATGAGACACGGTTCCTGGAGGAGCGGTTCATCGAGAGGGGCGGCATCTACGACTGGGGCAGCTCCTACCCCTACGACGTGCCAGATTACGCCTATCCTTACGACGTGCCAGATTATGCCTACCCCTATGACGTGCCTGATTATGCCTGA |
| >XP_042926382.1 | [*Chlamydomonas reinhardtii*](https://www.ncbi.nlm.nih.gov/Taxonomy/Browser/wwwtax.cgi?id=3055) | MLTSFQAGSGSLACQAPRRLPSPPQTAAVLHQQYCRSAQLALPPTVNWHLEPRCNYHCKFCFATFSDIPSSEVVKDADLLLAVPPLLAAAGVSKITFVGGEPLLHPLLPELLAAAKGAGLVTSLVSNGSLLTEDWLRRMQGHLDWIAFSVDASDDALHAALGRGTTRETGVTMPRHSSSSSSSTKNSSHRKPSGDGSSRGGATGSGTATSPAVGRGGGGGDVGGGVGTRGGHLARVERLWGVAQGLGYRLKLNTVVTAPCLGDVGGMVELVGRLRPERWKVFQVLPITGQTQPEHIAPLLTTEAQFADWVAAASVVQELYGVPLVPESNSHMHGSYAMLDARGRFYQDVNGEGYEYGPSIFDCWQGRLLESLALDSHAHPHQPRLAAAPSHDAAVGTSADDVHAAAMAVLMARPTSSPAAVGSGGVARGAAAGDGDGDGGNSNGAGPNARTQMQMEAGREAVMDAWGAVAGPAFRAEAFATRGGVYEWGQGGEALEEQQRAWGEAASSSGGAESDI**GSSYPYDVPDYAYPYDVPDYAYPYDVPDYA*** | ATGCTGACATCCTTCCAGGCAGGCTCTGGCAGCCTGGCCTGCCAGGCCCCCCGGAGACTGCCTTCTCCACCTCAGACCGCAGCCGTGCTGCACCAGCAGTACTGCAGGAGCGCCCAGCTGGCCCTGCCACCCACAGTGAACTGGCACCTGGAGCCACGCTGTAATTATCACTGCAAGTTCTGTTTTGCCACCTTCTCTGACATCCCCAGCTCCGAGGTGGTGAAGGACGCCGATCTGCTGCTGGCCGTGCCTCCACTGCTGGCCGCCGCCGGCGTGAGCAAGATCACCTTTGTGGGAGGAGAGCCACTGCTGCACCCACTGCTGCCTGAGCTGCTGGCCGCCGCCAAGGGAGCAGGACTGGTGACCAGCCTGGTGTCCAACGGCTCTCTGCTGACAGAGGACTGGCTGAGGCGCATGCAGGGCCACCTGGATTGGATCGCCTTTAGCGTGGACGCCTCCGACGATGCACTGCACGCCGCCCTGGGAAGGGGCACCACACGCGAGACCGGCGTGACAATGCCTCGGCACTCTAGCTCCTCTAGCTCCTCTACAAAGAATAGCTCCCACAGGAAGCCAAGCGGCGATGGCTCTAGCCGCGGCGGCGCCACCGGCTCTGGCACCGCCACAAGCCCTGCCGTGGGCAGGGGAGGAGGAGGAGGCGACGTGGGAGGAGGCGTGGGCACCCGCGGCGGCCACCTGGCCCGGGTGGAGAGACTGTGGGGCGTGGCCCAGGGCCTGGGCTACAGGCTGAAGCTGAACACCGTGGTGACAGCACCATGCCTGGGCGATGTGGGAGGAATGGTGGAGCTGGTGGGCCGGCTGAGACCAGAGCGGTGGAAGGTGTTCCAGGTGCTGCCTATCACCGGCCAGACACAGCCTGAGCACATCGCCCCACTGCTGACCACAGAGGCCCAGTTTGCAGACTGGGTGGCCGCCGCCAGCGTGGTGCAGGAGCTGTACGGCGTGCCACTGGTGCCCGAGTCCAACTCTCACATGCACGGCTCCTACGCCATGCTGGATGCCAGGGGCCGCTTCTATCAGGACGTGAATGGCGAGGGCTACGAGTATGGCCCATCCATCTTTGATTGTTGGCAGGGCCGGCTGCTGGAGTCCCTGGCCCTGGACTCTCACGCACACCCTCACCAGCCACGGCTGGCCGCCGCCCCAAGCCACGACGCAGCCGTGGGCACCTCCGCCGACGATGTGCACGCCGCCGCTATGGCCGTGCTGATGGCACGGCCAACATCCTCTCCAGCAGCAGTGGGCTCCGGAGGAGTGGCCAGAGGCGCTGCCGCCGGCGACGGCGATGGCGACGGAGGCAACTCTAATGGAGCAGGACCAAATGCAAGGACACAGATGCAGATGGAGGCCGGCAGAGAGGCCGTGATGGATGCATGGGGAGCAGTGGCAGGACCTGCCTTCCGGGCAGAGGCCTTTGCCACCAGAGGCGGCGTGTACGAGTGGGGACAGGGAGGAGAGGCCCTGGAGGAGCAGCAGAGGGCCTGGGGCGAGGCCGCCAGCTCCTCTGGAGGAGCAGAGAGCGACATCGGCAGCTCCTACCCTTATGATGTGCCAGACTACGCCTATCCCTACGATGTGCCTGACTATGCCTACCCCTACGACGTGCCCGACTATGCCTGA |
| >Vocar.0006s0432.1 | [*Volvox carteri f. nagariensis*](https://www.ncbi.nlm.nih.gov/Taxonomy/Browser/wwwtax.cgi?id=3068) | MTRRSFVSRLTRYAIDCSCAKGPLNPSWPPQGAVGTHHVCCHAGGHEPSTSYAYGIQSTGFHSVARRPSCARGFCSWPLTAAATSQTQSQEGNSSANWVLDPASAAEYDRRLREFLNASRALQDLPTARRDPPAPPPPPRPNRPLPPPPFHSFIAPAAAATIPAQDNTGFLVGDVATAVAAVASESTGSPAAASPTSPVPSTTSPPPGISPASPLSPPSLQSSSRVGLGPPEVASSSSSALQATAAAATARDRARAALRNVPAFTPARIGGCSSNSSNPSGPPANGCGGAAAAAALPRSEVSAALPQPVPAAPVLPAPPAPPHPASPTPVDWRGLLQRARQVLGSRQVAGAEMLTDKFSRIHTYLRISLTERCNLRCTYCMPDEGVALTPTPQLLTSREIMRLARIFVEAGVTKIRLTGGEPTLRRDITDLIRQLSSLRPMGLRSVAITSNGIVLARQLPELKEAGLTAVNISLDTLRGERFEQLARRPGHKRVLESIRTAVGLGYDPVKVNVVVMRGVNDDEVADFAALTRDQPINVRFIEYMPFDGNVWSDSKMVPYRELISRIQAAFPYTPLERLDDPAGEVAKNFRLWGHRGSVSFITSMTQHFCSDCNRLRLLADGNLKVCLFGAAEVSLRDAMRGGASDDDLRAIITAAVGRKRAAHAGMFELAASANRPMITIGG**GSSYPYDVPDYAYPYDVPDYAYPYDVPDYA*** | ATGACACGGCGGAGCTTCGTGAGCCGGCTGACCAGATACGCCATCGATTGCAGCTGTGCCAAGGGCCCACTGAATCCATCCTGGCCACCTCAGGGAGCAGTGGGCACACACCACGTGTGCTGTCACGCAGGAGGACACGAGCCCAGCACATCCTACGCCTATGGCATCCAGTCCACCGGATTCCACTCTGTGGCAAGGCGCCCCTCTTGCGCCAGAGGCTTTTGTAGCTGGCCTCTGACCGCCGCCGCCACCAGCCAGACACAGAGCCAGGAGGGCAACAGCTCCGCCAATTGGGTGCTGGACCCCGCCAGCGCCGCAGAGTATGACCGGAGACTGAGGGAGTTCCTGAACGCCTCCCGGGCCCTGCAGGATCTGCCTACCGCAAGGCGCGACCCACCAGCACCTCCACCACCTCCAAGGCCTAATAGGCCACTGCCACCTCCACCATTCCACAGCTTTATCGCACCTGCTGCCGCCGCCACAATCCCAGCCCAGGATAACACCGGCTTTCTGGTGGGCGACGTGGCAACCGCAGTGGCAGCAGTGGCCTCTGAGAGCACAGGCTCCCCAGCCGCCGCCAGCCCCACCTCTCCAGTGCCCAGCACCACATCCCCTCCACCAGGCATCTCTCCAGCCAGCCCTCTGTCCCCTCCATCTCTGCAGTCTAGCTCCCGCGTGGGACTGGGACCACCTGAGGTGGCCTCTAGCTCCTCTAGCGCCCTGCAGGCAACCGCTGCCGCCGCCACAGCCCGGGATAGAGCCAGGGCCGCCCTGAGGAATGTGCCAGCCTTTACCCCTGCAAGGATCGGAGGATGCTCCTCTAACAGCTCCAATCCTAGCGGCCCACCCGCCAACGGATGTGGAGGAGCCGCCGCCGCCGCCGCCCTGCCACGGAGCGAGGTGTCCGCCGCCCTGCCTCAGCCAGTGCCCGCCGCCCCAGTGCTGCCCGCCCCTCCAGCCCCCCCTCACCCAGCCTCTCCTACACCAGTGGATTGGAGAGGACTGCTGCAGCGGGCCAGACAGGTGCTGGGCTCCAGGCAGGTGGCAGGAGCCGAGATGCTGACCGACAAGTTCTCTCGCATCCACACATACCTGCGGATCAGCCTGACCGAGAGATGCAACCTGAGGTGCACATATTGTATGCCAGACGAGGGAGTGGCCCTGACCCCAACACCTCAGCTGCTGACCAGCCGCGAGATCATGAGACTGGCCAGGATCTTTGTGGAGGCCGGCGTGACCAAGATCAGGCTGACAGGAGGAGAGCCCACCCTGAGGAGAGACATCACAGACCTGATCCGGCAGCTGTCTAGCCTGAGACCTATGGGCCTGAGGAGCGTGGCCATCACCTCCAACGGAATCGTGCTGGCCAGGCAGCTGCCTGAGCTGAAGGAGGCCGGCCTGACAGCCGTGAATATCAGCCTGGATACCCTGAGGGGAGAGCGGTTCGAGCAGCTGGCCAGGCGCCCAGGACACAAGAGAGTGCTGGAGTCCATCAGGACAGCAGTGGGACTGGGATACGACCCAGTGAAGGTGAACGTGGTGGTCATGAGAGGCGTGAATGACGATGAGGTGGCCGATTTTGCCGCCCTGACCAGAGACCAGCCAATCAACGTGAGGTTCATCGAGTATATGCCCTTTGATGGCAACGTGTGGTCCGACTCTAAGATGGTGCCTTACCGCGAGCTGATCAGCAGAATCCAGGCCGCCTTCCCTTATACCCCACTGGAGAGGCTGGACGATCCAGCAGGAGAGGTGGCCAAGAACTTCCGGCTGTGGGGACACAGGGGCAGCGTGTCCTTCATCACCTCCATGACACAGCACTTTTGCTCTGATTGTAACAGACTGAGGCTGCTGGCCGACGGCAATCTGAAGGTGTGCCTGTTCGGAGCAGCAGAGGTGTCCCTGAGGGACGCCATGAGGGGAGGAGCCTCTGACGATGACCTGAGAGCCATCATCACAGCAGCAGTGGGAAGGAAGAGGGCAGCACACGCAGGAATGTTTGAGCTGGCCGCCTCCGCCAACAGGCCAATGATCACCATCGGCGGCGGCTCCTCTTACCCCTACGACGTGCCCGACTACGCCTATCCTTACGATGTGCCAGACTATGCCTACCCCTATGATGTGCCTGACTACGCCTGA |
| >XP_024378973.1 | [*Physcomitrium patens*](https://www.ncbi.nlm.nih.gov/Taxonomy/Browser/wwwtax.cgi?id=3218) | MRWVQWVALSSLRAARSLTTSSAPTGFGFHGYSGVSAVVCKQGSVVSFHALALEKEPQPEDERRDGEVDRHSNEKPRSDNLREKSSIEGVSPVRSSTIEADPRYQRRHAEEADRVSDMLTDSHGRRHNYLRISLTERCNLRCHYCMPAEGVELTPNSGLLSQEEIIRIASTFVAGGVDKIRLTGGEPSIRSDIEEICEQLRSLPGLQNLAMTSNGIILSRKLFRLQAAGLNQLNISLDTLVPAKFELLTRRKGHNKVLQSIDTALGLGFSPVKVNTVVMRGLNDDEILDFVEITRDRDINVRFIEFMPFDGNVWNPKKLVSYVEMMDTIKNKFPSIYRLKDHPTDTAKNFRVEGYLGTVSFITSMTQHFCSGCNRLRLMADGNLKVCLFGPSEVSLRDAVRSGMQESELQQVISDAVKRKKAAHAGMFELARTQNRPMIHIGG**GSSYPYDVPDYAYPYDVPDYAYPYDVPDYA*** | ATGAGATGGGTGCAGTGGGTGGCCCTGAGCAGCCTGAGGGCAGCAAGAAGCCTGACCACATCTAGCGCCCCTACCGGCTTCGGCTTTCACGGCTACTCCGGCGTGTCTGCCGTGGTGTGCAAGCAGGGCAGCGTGGTGTCCTTTCACGCCCTGGCCCTGGAGAAGGAGCCACAGCCTGAGGACGAGCGGAGAGATGGCGAGGTGGACAGACACAGCAACGAGAAGCCCAGGTCCGATAATCTGCGCGAGAAGTCCTCTATCGAGGGCGTGAGCCCCGTGCGGAGCAGCACCATCGAGGCCGACCCCAGATACCAGAGGCGCCACGCAGAGGAGGCAGATAGGGTGTCTGACATGCTGACCGATAGCCACGGCCGGAGACACAACTACCTGCGCATCTCCCTGACAGAGAGGTGCAATCTGCGCTGCCACTATTGTATGCCAGCCGAGGGCGTGGAGCTGACACCCAACAGCGGCCTGCTGTCCCAGGAGGAGATCATCAGGATCGCCTCCACCTTCGTGGCAGGAGGAGTGGACAAGATCAGGCTGACAGGCGGCGAGCCTTCTATCCGCAGCGACATCGAGGAGATTTGCGAGCAGCTGCGCTCTCTGCCAGGACTGCAGAACCTGGCCATGACCTCCAATGGCATCATCCTGTCTCGGAAGCTGTTTAGACTGCAGGCCGCCGGCCTGAACCAGCTGAATATCTCCCTGGATACCCTGGTGCCCGCCAAGTTCGAGCTGCTGACAAGGCGCAAGGGCCACAACAAGGTGCTGCAGAGCATCGACACCGCCCTGGGACTGGGCTTTTCCCCTGTGAAGGTGAACACAGTGGTCATGCGGGGCCTGAATGACGATGAGATCCTGGACTTCGTGGAGATCACACGGGACAGAGACATCAATGTGAGATTCATCGAGTTTATGCCTTTCGACGGCAACGTGTGGAATCCAAAGAAGCTGGTGTCTTACGTGGAGATGATGGATACCATCAAGAACAAGTTTCCCAGCATCTATCGGCTGAAGGACCACCCTACCGATACAGCCAAGAATTTCAGAGTGGAGGGCTACCTGGGCACAGTGTCTTTTATCACCAGCATGACACAGCACTTCTGCAGCGGCTGTAACAGGCTGCGCCTGATGGCCGACGGCAATCTGAAGGTGTGCCTGTTTGGCCCATCCGAGGTGTCTCTGAGGGATGCCGTGCGCTCCGGCATGCAGGAGTCTGAGCTGCAGCAGGTCATCAGCGACGCCGTGAAGAGGAAGAAGGCAGCACACGCAGGAATGTTCGAGCTGGCCCGGACCCAGAATAGACCCATGATCCACATCGGCGGCGGCTCTAGCTACCCCTACGACGTGCCCGATTACGCCTATCCTTACGACGTGCCAGATTATGCCTACCCCTATGACGTGCCTGATTACGCCTGA |
| >KMZ71380.1 | [*Zostera marina*](https://www.ncbi.nlm.nih.gov/Taxonomy/Browser/wwwtax.cgi?id=29655) | MAMRRRVMDLLRRESVFRITPNYLVGACSKAISQSTPATICEETNTSKSDMLVDSFGRQHTYLRISLTERCNLRCHYCMPSDGVDLTPNSSLLSSDEIIRVANLFVSSGVDKIRLTGGEPTIRKDIEDICSRLSNMEGLKSLGITTNGIVLARKLPKLREYGLNLLNISLDTLVPSKFEFMTRRKGHNKVLEAIDVAIDLGYNPVKVNCVIMRGINDDEICNFVELTKEKPINVRFIEFMPFDGNVWNVKKLVSYAEMFDIVTKRFEGVKRCQDHPSDTAKNFSIDGHRGTISFITSMTEHFCGGCNRLRLLADGNFKVCLFGPSEVSLRDPIRSGTDDDGLRDIIGAAVKRKKASHAGMFDIAKTANRPMIHIGG**GSSYPYDVPDYAYPYDVPDYAYPYDVPDYA*** | ATGGCCATGCGGCGGAGAGTGATGGATCTGCTGCGCCGGGAGAGCGTGTTTCGGATCACCCCCAACTACCTGGTGGGCGCCTGCTCCAAGGCCATCTCTCAGAGCACCCCTGCCACAATCTGTGAGGAGACCAATACATCCAAGTCTGATATGCTGGTGGACTCCTTCGGCAGACAGCACACCTACCTGAGGATCTCTCTGACAGAGAGATGCAACCTGAGGTGCCACTATTGTATGCCCAGCGATGGCGTGGACCTGACCCCTAACAGCTCCCTGCTGTCTAGCGATGAGATCATCAGGGTGGCCAATCTGTTCGTGAGCAGCGGAGTGGACAAGATCAGGCTGACCGGAGGAGAGCCAACAATCCGGAAGGACATCGAGGACATCTGTAGCAGACTGTCCAACATGGAGGGCCTGAAGTCCCTGGGCATCACCACAAATGGCATCGTGCTGGCCCGCAAGCTGCCAAAGCTGCGGGAGTACGGCCTGAACCTGCTGAATATCAGCCTGGACACCCTGGTGCCCTCCAAGTTCGAGTTTATGACAAGAAGGAAGGGCCACAACAAGGTGCTGGAGGCCATCGATGTGGCCATCGACCTGGGCTATAACCCTGTGAAAGTGAATTGCGTGATCATGCGGGGCATCAACGACGATGAGATTTGCAATTTTGTGGAGCTGACCAAGGAGAAGCCTATCAATGTGAGATTCATCGAGTTTATGCCATTCGATGGCAACGTGTGGAATGTGAAGAAGCTGGTGAGCTACGCCGAGATGTTTGACATCGTGACAAAGAGATTCGAGGGCGTGAAGAGGTGCCAGGATCACCCATCTGACACCGCCAAGAACTTCAGCATCGATGGCCACAGGGGCACAATCTCCTTTATCACCTCTATGACAGAGCACTTCTGCGGAGGATGTAACAGGCTGAGGCTGCTGGCCGACGGAAACTTCAAGGTGTGCCTGTTCGGCCCATCTGAGGTGAGCCTGCGCGATCCCATCCGGTCTGGCACCGACGATGACGGACTGAGGGACATCATCGGAGCAGCAGTGAAGCGCAAGAAGGCCTCCCACGCCGGCATGTTCGACATCGCCAAGACAGCCAATCGCCCTATGATCCACATCGGCGGCGGCAGCTCCTACCCCTATGATGTGCCTGACTACGCCTATCCATACGATGTGCCCGACTATGCCTACCCTTATGATGTGCCAGACTATGCCTGA |
| >KAF5832982.1 | [*Dunaliella salina*](https://www.ncbi.nlm.nih.gov/Taxonomy/Browser/wwwtax.cgi?id=3046) *E* | MLQMRSLERLAISLRAFRQSASCSPPSWAAAPCASDIRGVQSDGDIGAPSSSGRDAGGTHQNWTLTWALRQGSPGTANSNTQDDRPWFLRLEPSTARHAHFSTAAPVPKPRRQTRLRIEDVPMFQIERKDSSKRDSSNRDPKLQPSPSHAGLDWREMLARARASQEAESTVQGGPAMVTDTFSRVHTYLRISLTEKCNLRCQYCMPAEGIALTPNQRLLTTQEIMRLTRLFVEAGINKVRLTGGEPTLRPDLVDLCHQLKVLPGLETIAITTNGITLSRNLPALQEAGLSAINISLDTLRPERFEVMSRRPGHDKVMRSIDKALSMGYNPVKLNVVVMRGVNDDELNDFVALTRDHPINVRFIEYMPFDGNVWSDKKMVPYREMMARVREALQQQHQLDQRQQQQQQQQQELSPVETAASNSHQSSSSSSSSSCSEHNQLLERLADPNGEVAKNFRVPGHAGIISFVTSMTSHFCGECNRLRLMADGNLKVCLFGASEVSLRDAMRGGATDEDLRLIIGAAVRRKKAKHAGMFEIAATQNRPMITIGG**GSSYPYDVPDYAYPYDVPDYAYPYDVPDYA*** | ATGCTGCAGATGCGGTCCCTGGAGAGACTGGCCATCTCTCTGAGGGCCTTTCGCCAGAGCGCCTCCTGCTCTCCACCTAGCTGGGCTGCCGCCCCTTGTGCCAGCGACATCAGGGGAGTGCAGTCCGACGGCGACATCGGCGCCCCAAGCTCCTCTGGCAGAGACGCAGGAGGCACCCACCAGAACTGGACCCTGACATGGGCACTGAGGCAGGGCAGCCCAGGCACCGCCAACTCCAATACACAGGACGATCGGCCCTGGTTCCTGAGACTGGAGCCTTCTACCGCCAGACACGCCCACTTTAGCACAGCAGCACCAGTGCCCAAGCCTCGGAGACAGACAAGGCTGCGCATCGAGGATGTGCCCATGTTCCAGATCGAGCGCAAGGACAGCTCCAAGAGGGATTCTAGCAATCGCGACCCTAAGCTGCAGCCAAGCCCATCCCACGCAGGCCTGGATTGGAGGGAGATGCTGGCCCGGGCCAGAGCCTCTCAGGAGGCAGAGAGCACCGTGCAGGGAGGACCTGCTATGGTGACCGACACATTTTCCCGGGTGCACACCTACCTGAGAATCTCTCTGACAGAGAAGTGCAACCTGCGCTGCCAGTATTGTATGCCAGCAGAGGGAATCGCCCTGACCCCAAATCAGCGGCTGCTGACCACACAGGAGATCATGCGGCTGACAAGACTGTTCGTGGAGGCCGGCATCAACAAGGTGCGGCTGACCGGAGGAGAGCCTACACTGAGACCAGACCTGGTGGACCTGTGCCACCAGCTGAAGGTGCTGCCAGGCCTGGAGACCATCGCCATCACCACAAACGGCATCACACTGTCCAGGAATCTGCCCGCCCTGCAGGAGGCAGGACTGTCCGCCATCAATATCTCTCTGGACACCCTGAGGCCTGAGCGGTTCGAAGTGATGAGCAGGCGCCCAGGCCACGATAAAGTGATGCGCTCTATCGACAAGGCCCTGAGCATGGGCTACAACCCCGTGAAGCTGAATGTGGTGGTCATGCGGGGCGTGAACGACGATGAGCTGAATGATTTCGTGGCCCTGACAAGGGACCACCCCATCAACGTGCGCTTCATCGAGTACATGCCTTTTGATGGCAACGTGTGGTCCGACAAGAAGATGGTGCCTTATAGAGAGATGATGGCCAGGGTGCGCGAGGCCCTGCAGCAGCAGCACCAGCTGGATCAGAGGCAACAACAGCAACAACAGCAGCAGCAGGAGCTGTCCCCAGTGGAGACCGCAGCCTCTAACAGCCACCAGTCCTCTAGCTCCTCTAGCTCCTCTAGCTGCAGCGAGCACAATCAGCTGCTGGAGAGGCTGGCCGACCCAAACGGAGAGGTGGCCAAGAATTTCCGCGTGCCCGGACACGCAGGAATCATCAGCTTCGTGACCTCCATGACATCTCACTTTTGCGGCGAGTGTAACCGGCTGAGACTGATGGCCGATGGCAATCTGAAGGTGTGCCTGTTTGGAGCCAGCGAGGTGTCCCTGAGGGACGCAATGAGGGGAGGAGCAACCGACGAGGATCTGAGACTGATCATCGGCGCCGCCGTGCGGAGAAAGAAGGCCAAGCACGCCGGCATGTTCGAGATCGCCGCCACCCAGAACAGGCCCATGATCACAATCGGCGGCGGCTCCTCTTACCCCTATGACGTGCCTGATTACGCCTATCCATACGACGTGCCCGATTATGCCTACCCTTATGACGTGCCAGATTATGCCTGA |

**Table 2. Primers used in this study**

| **Name of primer** | **Sequence (5’-3’)** | **Purpose** |
| --- | --- | --- |
| TuMV/PVX-GFP qPCR-F | gaacttttcactggagttgtcccaat | Forward primer of qPCR for GFP sequence of TuMV-GFP and PVX-GFP |
| TuMV/PVX-GFP qPCR-R | ggaacaggtagttttccagtagtgc | R primer of qPCR for GFP sequence of TuMV-GFP and PVX-GFP |
| PP2A-F | GACCCTGATGTTGATGTTCGCT | Forward primer of qPCR for Protein phosphatase sequence (internal control) |
| PP2A-R | GAGGGATTTGAAGAGAGATTTC | Reverse primer of qPCR for Protein phosphatase sequence (internal control) |
